# Hydraulic mechanisms of the uneven enrichment of soil organic carbon in sediments during rain-induced overland flow

**DOI:** 10.1371/journal.pone.0262865

**Published:** 2022-02-22

**Authors:** Lin Liu, Zhongwu Li, Panpan Jiao

**Affiliations:** 1 State Key Laboratory of Soil Erosion and Dryland Farming on the Loess Plateau, Institute of Soil and Water Conservation, CAS and MWR, Yangling, Shaanxi Province, PR China; 2 College of Geography and Environment, Shandong Normal University, Jinan, Shandong, PR China; 3 College of Resources and Environmental Sciences, Hunan Normal University, Changsha, Hunan, PR China; Institute of Earth and Environment, Chinese Academy of Sciences, CHINA

## Abstract

Organic carbon (OC) can be unevenly enriched in different-sized sediment particles under low-intensity, rain-induced overland flows, but its hydraulic mechanisms are not completely understood. Hence, in this study, the hydraulic transport mechanisms of unevenly enriched OC between different-sized sediment particles were investigated through simulated rainfall experiments at gradients of 5°, 10°, and 15° and typical regional rainfall intensities of 45, 90, and 120 mm h^−1^. Results showed that the critical flow velocity of aggregate transport through loess soil was approximately 0.08 m s^−1^. When the flow velocity was larger than this critical value, the aggregate loss amount increased quickly and exponentially. Flow velocities lower than 0.08 m s^−1^ were determined to be essential conditions for uneven OC enrichment between sediment particles. At such velocities, even when the runoff depth was greater than 0.0018 m, the enrichment ratio of soil organic carbon (SOC; *ER*_*oc*_) values in all size classes of sediment particles was larger than 1.0. Small runoff depths caused preferential OC enrichment in silt and clay, whereas large runoff depths promoted OC enrichment in the >0.25 mm size class of sediment particles. The critical flow velocity and transport way differ between these high-OC-concentration clay and silt and large light organic particles. The interaction between flow velocity and runoff depth on *ERocs* in <0.05 mm particles was larger than that of >0.05 mm particles. Under the transport limit erosion, the flow velocity and stream power positively correlated with uneven *ER*_*ocs*_ in different size sediment particles through distinct laws. Slope and rainfall intensity could not be ignored in predicting uneven OC enrichment in sediments by interacting with hydraulic factor and effecting aggregate stripping, respectively. Hydraulic factors mainly affected the uneven OC enrichment by controlling particle selective detachment and transport process. Owing to the different hydraulic mechanisms of OC enrichment in different size particles, the obtained regression functions for uneven OC enrichment could be divided into two types. One was for calculating the OC concentrations in sediment particles with sizes of <2 mm (*R*^*2*^ > 0.844, *P* < 0.005), and the other was for calculating the OC concentrations in large macroaggregates (>2 mm; *R*^*2*^ = 0.805, *P* < 0.005). The findings provide an important reference for understanding SOC transport mechanisms and its mineralization potential under the effect of water erosion and improving SOC dynamic models.

## 1. Introduction

Soil is an important carbon pool in terrestrial ecosystems [1]. The global soil carbon pool of 2500 gigatons (Gt) includes about 1550 Gt of soil organic carbon (SOC). The soil SOC pool is 2 times the size of the atmospheric pool (760 Gt) and 3 times the size of the biotic pool [2]. Soil water erosion considerably affects the soil carbon stock, which influences atmospheric CO_2_ concentration [3, 4] and decreases soil productivity [5–9]. However, due to the complexity of soil organic carbon (SOC) loss, transport, and distribution, the role of agricultural soils as carbon sinks or sources during water erosion remains controversial [10]. As an important link in the soil carbon cycle, the horizontal transport mechanism of SOC caused by rain-induced runoff should be clarified to solve the aforementioned controversy. Many researchers have studied SOC loss, which has been mainly explained based on the soil erosion mechanism [8, 11]. However, the transport mechanisms of SOC and sediments differ because SOC can be easily enriched between different-sized sediment particles, especially for labile SOC fractions with low densities [12]. Therefore, the related mechanisms of SOC enrichment in different size classes of sediment particles should be explored comprehensively.

Previous studies have found that because of aggregate breakdown and selective transport of SOC fractions, the SOC concentration in sediments varies during erosion [13–15]. When the runoff erosive power is low enough, SOC can also be unevenly enriched between different size classes of sediment particles in soils with high SOC and aggregate contents [16, 17]. This uneven enrichment is due to the macroaggregates being broken down into microaggregates and even smaller particles with different organic carbon (OC) concentrations [18]. During this process, raindrop impact produces sediments with finer sizes than the original soil [19] and changes the OC concentration for all size classes of particles [16, 20, 21]. Given that light particles with high OC concentration are easily transported, large amounts of low-density OC are unevenly enriched in sediments and can be decomposed by microorganisms easily [11, 12, 14, 17]. However, only a few studies have examined the mechanisms of uneven SOC enrichment between sediment particles. Further studies on the quantitative relationships between uneven SOC enrichment ratio (*ER*_*oc-i*_) and hydraulic factors are necessary.

The hydraulic mechanisms of SOC fraction transport associated with sediment size distribution and runoff selective transport provide important theoretical support for tracing SOC during erosion and after deposition. Aside from carbon loss characteristics [22–24] and their influencing factors, such as the cultivation method and soil crust [25–28], current studies on SOC loss under water erosion have focused on the quantitative relationships between sediment loss and SOC enrichment in sediments. Studies that focused on the hydraulic mechanisms of uneven OC enrichment are rare. The runoff hydraulic mechanisms of sediments or OC loss is the theoretical basis of model soil erosion and SOC dynamics. The fractions of various sizes of particles are predicted during sediment particle sorting in the Water Erosion Prediction Project (WEPP) model on the basis of physical hydraulic mechanisms and sediment delivery features [29]. However, the erosion mechanisms of loessal soil on the Loess Plateau are distinct from those of other soils. Although the OC enrichment ratio (*ER*_*oc*_) in sediments is usually logarithmically related to sediment loss [30], the SOC loss sub-model in an SOC dynamic model, such as the CENTURY model, usually simulates the amount of soil loss in accordance with the Revised Universal Soil Loss Equation [19, 31, 32]. The *ER*_*oc*_ in sediments, an important index for calculating SOC loss [33], is rarely considered in current SOC dynamic models. Thus, relational models concerning the detailed calculation of uneven SOC enrichment in different size classes of sediment particles are necessary for the possible improvement of the SOC model. This study aims to (i) investigate the rain-induced flow hydraulic features of uneven SOC enrichment in sediment particles; (ii) clarify the hydraulic mechanisms of uneven SOC enrichment, and (iii) determine the quantitative relationships between runoff hydraulic characteristics and the uneven *ER*_*oc-i*_ of different size classes of sediment particles. This study provides an important reference for further understanding the changes in SOC at sites under water erosion and improving SOC dynamic models.

## 2. Methods and materials

### 2.1 Simulated rainfall experiments

**Fig 1:**
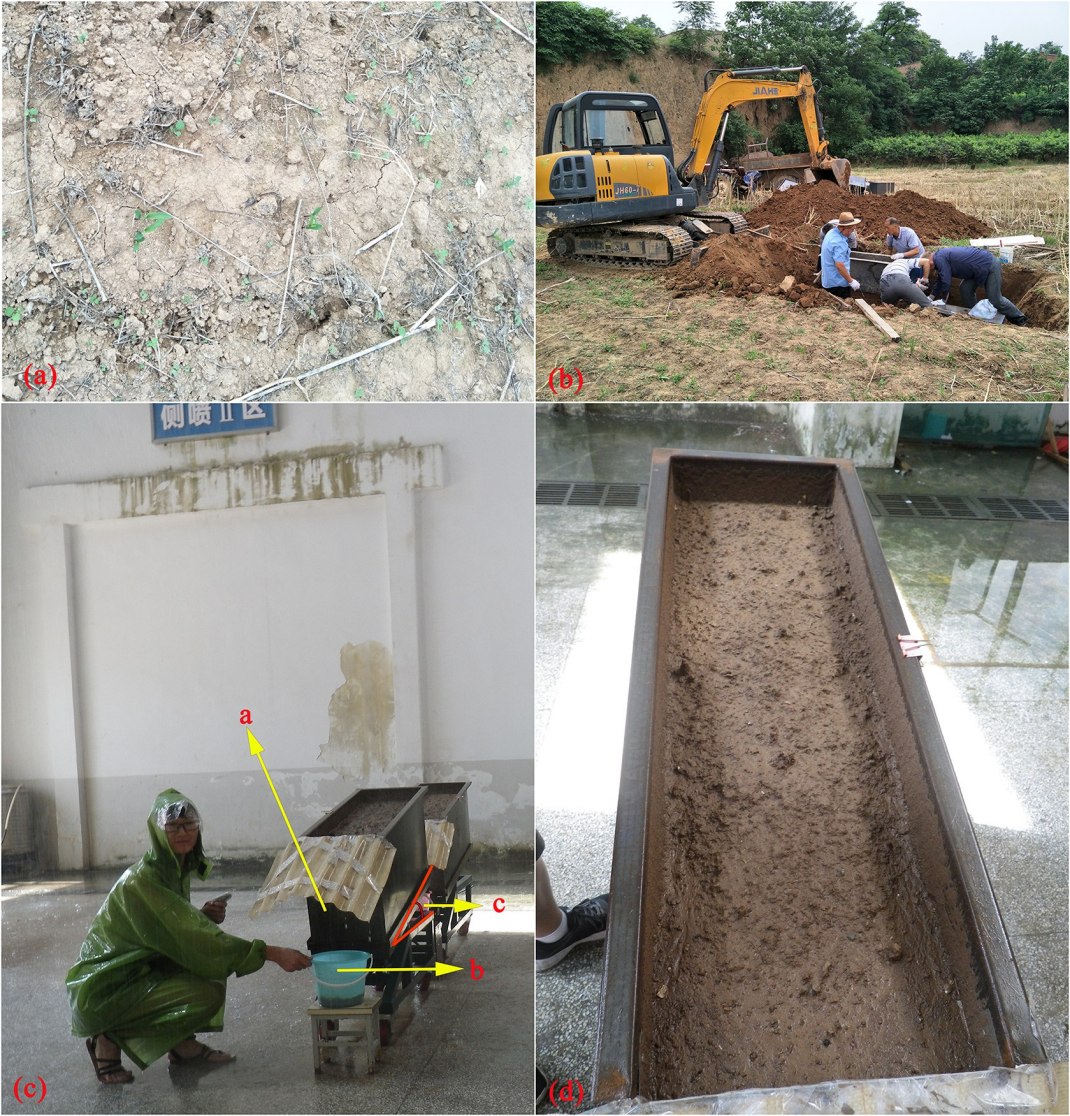
The sampling site ((a) and (b)) and experimental plot ((c); a, outlet for collecting runoff; b, sample bucket to collect samples and c, slope) and soil surface suffering rain-induced soil erosion (d).

Rainfall experiments were performed at the Institute of Soil and Water Conservation of the Ministry of Water Resources and Chinese Academy of Sciences in Yangling, Shaanxi, China. The tested loess soil characterized by high aggregate and SOC contents were collected from a cultivated field in Yangling (34° 16′ N, 108° 4′ E) on the Loess Plateau. The sample site has an altitude of 490–524 m above sea level and a semi-humid continental monsoon climate due to its location in a warm temperate zone. It had been cropped with a six-year rotation that included maize (*Zea mays* L.) and rapeseed (*Brassica campestris* L.). Ammonium bicarbonate N was applied in July at a fertilizer rate of 270 kg ha^-1^ after rapeseed was harvested. Superphosphate P was applied in November at a fertilizer rate of 100 kg ha^-1^. Reduced tillage was conducted, and no moldboard plowing was performed to sow seeds. The detailed properties of the tested loess soil are shown in Table 1. The soil was sampled before summer maize was sown (Fig 1a). To obtain undisturbed natural soil, the soil was dug out two days after rainfall to maintain its original shape and pores in soil mass. During the soil sampling process, the soil around the sampled soil was first dug away, and the sampled soil was placed in an iron collection box without a bottom or cover (Fig 1b). The soil and box were moved and covered with a bottom cap with through holes. The undisturbed soil was placed on a soil pan (1 m × 0.35 m × 0.40 m) as soon as it was collected, and a total of 27 soil samples were obtained. To reduce the differences among the repeated studies, the surface of the original soil was not disturbed before rainfall, and the soil pan was wetted from the top with water applied as mist to be saturated prior to performing the experiments. Then, the soil was set aside for one night to achieve some semblance of a natural slope. A lateral sprinkler rainfall simulator device was used. The nozzles of the simulator were installed 16 m above the ground, and a uniform rainfall intensity greater than 0.85 was used to simulate natural rainfall. The rainfall intensity and slope were varied for each rainfall experiment. Three typical slope gradients representing slight, gentle, and steep slopes (i.e., 5°, 10°, and 15°, respectively) and three typical rainfall intensities (i.e., 45, 90, and 120 mm h^−1^) in the sub-humid climate regions of China were selected [34–36]. Each treatment was repeated thrice. During the rainfall simulation process, runoff was collected at the slope outlet every 3 min (Fig 1c and 1d). Approximately 150 mL of each runoff sample was collected and placed in a beaker for sediment particle size distribution measurements. The changes in the slope of the eroded soil were recorded with a camera during the rainfall simulation process. The flow velocity in the middle of each plot was measured at 3 min intervals through the dye tracing method [37]. During runoff initiation, given that flow was not obvious in several cases, flow velocity was measured when flowing water became evident. Runoff depth was measured with a millimeter ruler, and all rainfall experiments were performed for 60 min.

**Table 1:** Basic characteristics of the original soil used in our study.

Property	Clay (%)	Fine silt (%)	Coarse silt (%)	Fine sand (%)	Coarse sand (%)	SOC_cs_	SOC_micro_	SOC_smacro_	SOC_lmacro_	SOC of the original soil (g kg^−1^)
Mean values (%)	26.3	38.1	28.7	5.6	1.3	5.33	10.20	7.76	13.54	5.68
Standard errors	2.9	0.5	1.0	0.6	0. 6	0.02	0.55	1.09	2.50	0.20

Soil texture is classified on the basis of the USDA soil classification system.

SOC_cs_: The SOC concentration in < 0.05 mm silt and clay particles of the original soil; SOC_micro_: The SOC concentration in 0.05–0.25 mm microaggregates of the original soil; SOC_smacro_: The SOC concentration in 0.25–2 mm small macroaggregates of the original soil; SOC_lmacro_: The SOC concentration in > 2 mm large macroaggregates of the original soil; The water stable aggregates were separated following the modified method described by Six et al. (1998) [38].

### 2.2 Runoff sample measurement

The effective sediment particle size distributions of the samples were measured immediately with a Malvern Mastersizer 2000 laser diffraction device (Malvern Instruments Ltd., UK) without chemical and physical dispersion. After that, organic matter in the sediments was removed by using H_2_O_2_, followed by chemical dispersion using sodium hexametaphosphate and ultrasonic dispersion to enable the accurate measurement of dispersed particle size distributions. Water stable aggregates were collected by using a modified wet-sieving method with a series of three sieves (i.e., 2, 0.25, and 0.053 mm) [39, 40]. The SOC concentrations in each size water stable aggregates (i.e., >2, 0.25–2, 0.053–0.250, and <53 mm) were measured through the dichromate oxidation method [41]. The SOC concentration in each water-stable aggregate size was expressed on a sand-free aggregate basis. *ER*_*oc-i*_ was defined as follows:

$$ERoc-i=\frac{C_{i}}{C_{o-i}},$$ (1)

where *C*_*i*_ is the SOC concentration in the ith water-stable aggregate size (g kg^−1^) and *C*_*o-i*_ is the SOC concentration in the ith water-stable aggregate size of original soil (g kg^−1^). The contribution of aggregate breakdown to SOC enrichment in the sediments (*Ca*) were determined as follows:

$$Ca=\frac{\sum_{1}^{i=4}\left(C_{i}-C_{i-o}\right)\times \omega_{i}}{C_{soc}-C_{o}}\times 100\text{\%,}$$ (2)

where *ω*_*i*_ is the weight fraction of the *i*th size class of sediment particles (%), *C*_*soc*_ is the SOC concentration in the sediments (g kg^−1^), and *C*_*o*_ is the SOC concentration in the original soil (g kg^−1^). Aggregate content was represented by two parameters: differences between the percentages of effective and dispersed sediment size classes (*Der*) and effective/dispersed particle size distribution (*E/D*). They can be determined as follows:

$$Der_{i}=E_{i}-D_{i},$$ (3)

$${\left(\frac{E}{D}\right)}_{i}=\frac{E_{i}}{D_{i}},$$ (4)

where *E*_*i*_ is the effective percentage and *D*_*i*_ is the dispersed percentage of the *i*th size class of sediments. In our study, the errors in the measurements obtained by the laser diffraction device and wet-sieving method for aggregate content detection in all size classes in the sediments were ignored. Given that only SOC concentrations in the sediments are discussed in our paper, these errors have no considerable effect on the correlation between aggregate content and SOC concentration in the sediments.

### 2.3 Hydraulic runoff characteristics

Runoff depth, shear stress, and stream power were determined from the flow velocity and runoff rate as follows:

$$d=\frac{q}{v},$$ (5)

$$\tau =\rho_{o}gds,$$ (6)

$$\omega =\rho_{o}gqs,$$ (7)

where *q* is the runoff discharge per unit slope width (m^2^ s^−1^), *d* is the runoff depth (m), *v* is the runoff velocity (m s^−1^), *τ* is the runoff shear stress (Pa), *ρ*_*o*_ is the runoff density (kg m^−3^) and assumed to have a constant value of 1000 kg m^−3^, *g* is the gravitational constant (9.8 m s^−2^), *s* is the slope gradient (m m^−1^), and *ω* is the runoff stream power (Ω; W m^−2^). Inevitable measurement errors were ignored during the calculation of the runoff hydraulic parameters.

### 2.4 Data analysis

Contour maps of *ER*_*oc*_ (<0.05 mm), *ER*_*oc*_ (0.25–0.05 mm), *ER*_*oc*_ (2–0.25 mm), and *ER*_*oc*_ (>2 mm) in the sediments were drawn to analyze the effects of rainfall intensity, runoff depth, and flow velocity on the transport regulation of OC in the aggregates of each size class. The *Der* and *E/D* values of the aggregates were used to represent the amounts of aggregates in the sediments. Our study is not based on the sand-free assumption, and changes in effective and dispersed sediment size distributions were used to represent aggregate contents. Moreover, *ER*_*oc-i*_ represents the ratio of the OC concentration of sediment particles in one size class to the OC concentration of soil particles in the same size class. Analyses and visualization were carried out using IBM SPSS Statistics 19.0 and Software Origin 8.0, respectively.

## 3. Results

### 3.1 Runoff hydraulic characteristics during erosion

**Fig 2:**
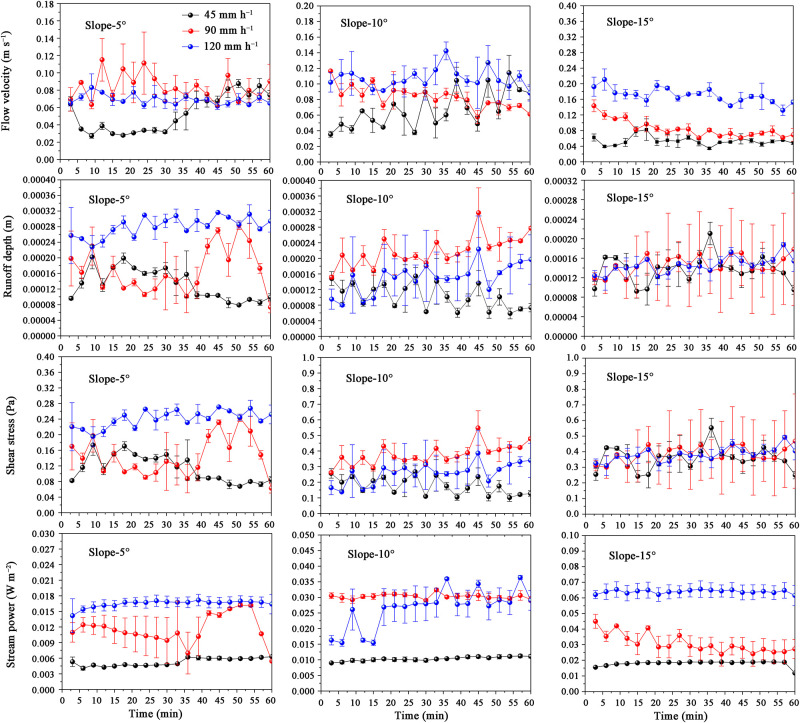
Changing trend of flow velocity, runoff depth, shear stress, and stream power during the erosion process.

Flow velocity decreased over time when the rainfall intensity was 90 or 120 mm h^−1^, increased with rainfall intensity from 0.053 m s^-1^ to 0.170 m s^-1^, and changed minimally with the slope (Fig 2 and Table 2). Flow velocity fluctuated considerably at low rainfall intensities (e.g., 45 mm h^−1^) or slopes (e.g., 5°; Fig 2). Runoff depth increased over time when the rainfall intensity was 90 or 120 mm h^−1^. However, runoff depth fluctuated widely when the rainfall intensity was 45 mm h^-1^ on the slope that was below or equal to 10°. With the decrease in slope, the effect of rainfall intensity on runoff depth increased. The runoff depths ranged from 0.00010 m to 0.00028 m. During erosion, shear stress showed a similar pattern with runoff depth, especially on the 5° slope. Low shear stress was observed at 45 mm h^−1^. For the average shear stress during rainfall, the largest values of shear stress were obtained at 90 mm h^−1^. The average shear stress during rainfall increased more obviously with slope than with rainfall intensity. At 90 or 120 mm h^−1^, shear stress increased over time, especially on the 5° slope. Meanwhile, stream power increased significantly with rainfall intensity and slope and minimally changed over time during erosion. Thus, the *ER*_*oc*_ values of the sediment particles in each size class and the total *ER*_*oc*_ values in the sediments decreased with increasing stream power (Table 3).

**Table 2:** Runoff hydraulic characteristics (runoff rate, flow velocity, runoff depth, shear stress and stream power) under different rainfall intensity and slope.

Treatment (rainfall intensity-slope)	Runoff rate (ml s^-1^)	Flow velocity (m s^-1^)	Runoff depth (m)	Shear stress (Pa)	Stream power (W m^-2^)
45–5	2.15±0.09	0.052±5.5E-3	1.33E-4±1.27E-5	0.11±0.01	0.01±2.10E-4
45–10	2.09±0.11	0.067±9.7E-3	1.03E-4±2.01E-5	0.12±0.03	0.01±5.08E-4
45–15	2.4±0.03	0.054±7.6E-3	1.37E-4±1.74E-5	0.36±0.05	0.02±2.17E-4
90–5	5.85±0.65	0.084±1.4E-2	1.69E-4±2.95E-5	0.14±0.03	0.01±2.20E-3
90–10	6.14±0.18	0.083±6.9E-3	2.19E-4±2.06E-5	0.34±0.04	0.03±8.77E-4
90–15	5.85±0.37	0.085±7.6E-3	1.46E-4±6.39E-5	0.38±0.17	0.03±5.70E-3
120–5	6.73±0.47	0.069±5.0E-3	2.81E-4±1.60E-5	0.24±0.01	0.02±1.14E-3
120–10	5.28±0.46	0.107±1.5E-2	1.51E-4±4.75E-5	0.26±0.08	0.03±3.41E-3
120–15	8.53±0.62	0.17±1.2E-2	1.46E-4±1.25E-5	0.38±0.03	0.06±4.64E-3

**Table 3:** Soil loss, SOC concentration in sediment particles, and OC enrichment ratios in the sediment particles (*ERocs*) for all treatments.

Treatment (rainfall intensity-slope)	Average sediment concentration (kg L^−1^)	Total sediment loss (kg)	*ERoc* of <0.05 mm	*ERoc* of 0.05–0.25 mm	*ERoc* of 0.25–2 mm	*ERoc* of > 2 mm	*Ca* (%)	SOC concentration in sediments	*ERoc* in sediments
45–5	0.006	0.05	2.17	3.51	none	none	94.2	13.43±1.98	2.36±0.15
45–10	0.020	0.16	1.42	1.44	2.55	1.37	73.57	11.12±1.01	1.96±0.09
45–15	0.024	0.21	1.54	1.44	1.60	1.00	78.13	10.30±0.30	1.81±0.03
90–5	0.012	0.21	1.41	1.46	2.58	none	84.18	10.14±0.77	1.79±0.08
90–10	0.039	0.85	1.26	1.32	1.52	1.16	62.38	8.88±0.65	1.56±0.07
90–15	0.024	1.01	1.22	1.25	1.41	1.00	53.27	9.07±0.85	1.60±0.09
120–5	0.030	0.74	1.48	2.26	2.46	0.91	86.94	10.93±1.52	1.92±0.14
120–10	0.720	9.43	1.17	1.11	1.13	0.68	73.24	6.58±0.09	1.16±0.01
120–15	0.067	2.08	1.24	1.23	1.40	0.97	57.23	8.63±0.32	1.52±0.04

### 3.2 Relationships between hydraulic factors and transport of sediment particles in all size classes

**Fig 3:**
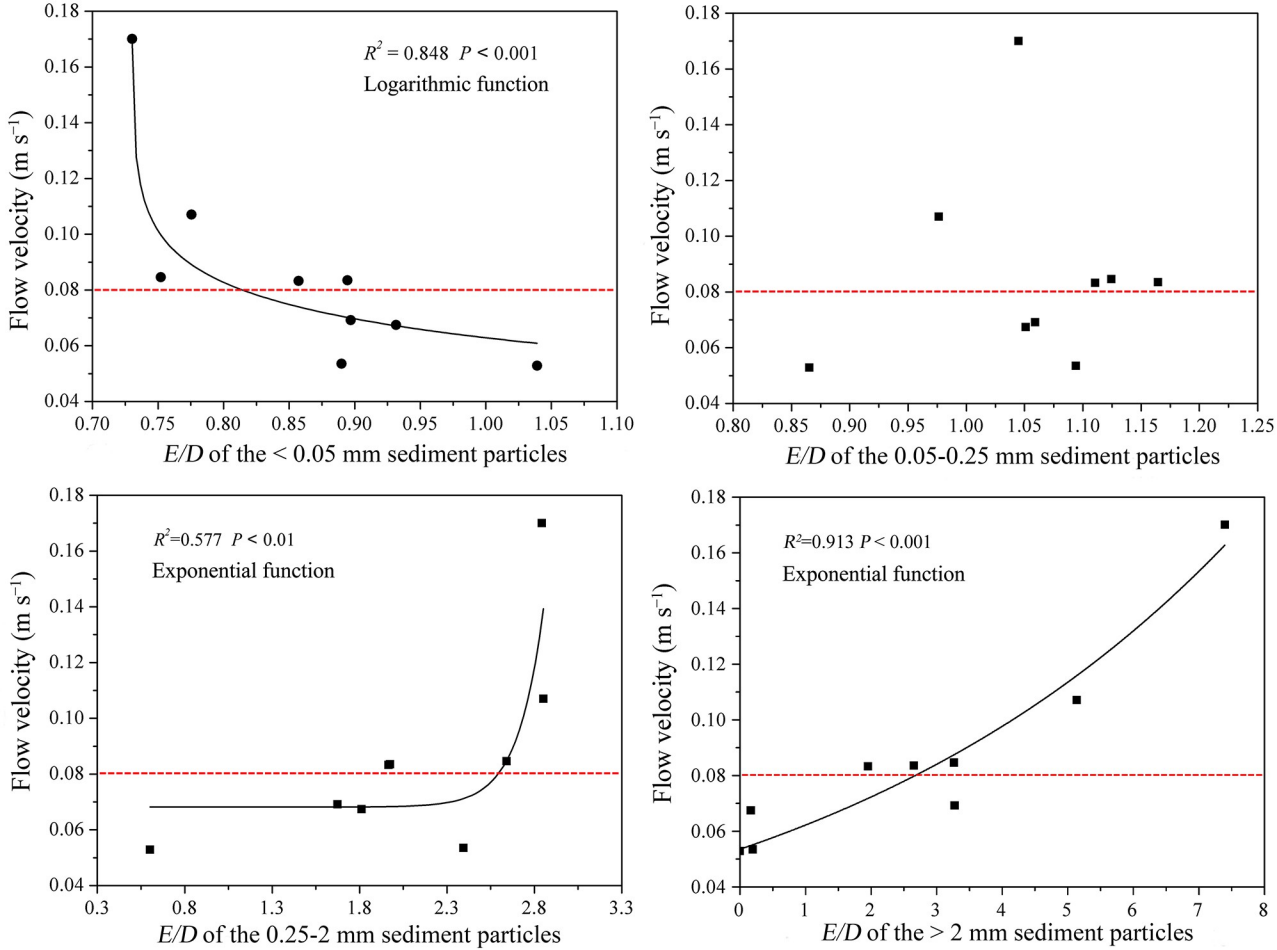
Relationships of flow velocities and effective/dispersed (*E/D*) particle size distribution.

To investigate the quantitative relationship between hydraulic factors and the amounts of sediment aggregates, we established the regression relationships of flow velocity with *E/D* (Fig 3). Nonlinear regression analysis revealed that flow velocity was logarithmically correlated with the *E/D* of the <0.02 mm sediment particles (*R*^*2*^ = 0.848, *P* < 0.001). When large amounts of clay and fine silt wrapped onto the aggregates, low *E/D* values were obtained for the <0.02 mm sediment particles. Thus, a positive correlation existed between flow velocity and the amount of clay and fine silt wrapped onto the aggregates. An exponential correlation was also observed between flow velocity and the *E/D* values of the 0.05–0.25 mm sediment particles (*R*^*2*^ = 0.577, *P* < 0.001), indicating positive relationships between flow velocity and the amount of microaggregates in the sediment particles. The flow velocity and *E/D* values of coarse sand (> 0.25 mm) showed a positive exponential correlation (*R*^*2*^ = 0.913, *P* < 0.001), which means that flow velocity and the amount of macroaggregates (> 0.25 mm) in the sediment particles had a positive linear correlation. However, flow velocity had no direct relationship with the *E/D* values of coarse silt (0.02–0.05 mm). Altogether, these results show that flow velocity had varied close relationships with the transport of aggregates in the sediment particles of different size classes. The effects of flow velocity were unchanged in the macroaggregates but exhibited several changes in the clay and fine silt wrapped onto the aggregates and microaggregates. A critical flow velocity of approximately 0.08 m s^−1^ existed for the transport of clay and fine silt wrapped onto aggregates and microaggregates in the loess soil.

**Fig 4:**
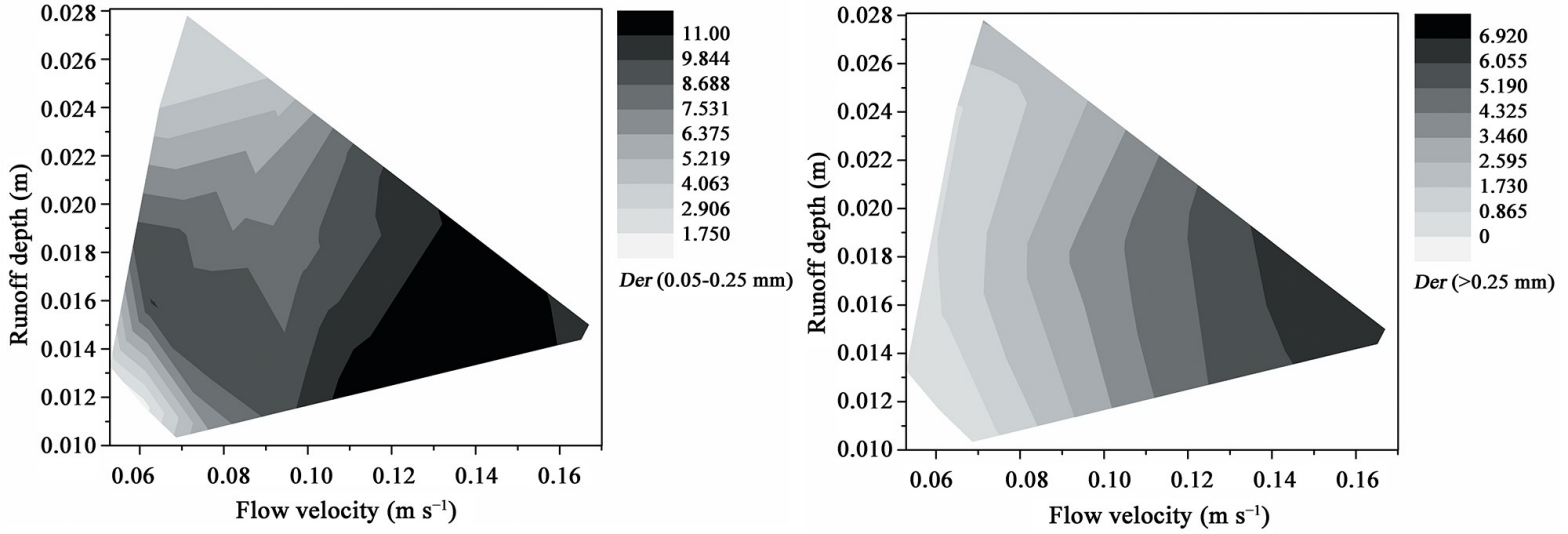
Effects of runoff depth–flow velocity interaction on the differences in the percentages of effective to dispersed (*Der*; %) fine sand (0.05–0.25 mm) or coarse sand (>0.25 mm). *Der* (0.05–0.25 mm) is the difference in the percentages of effective to dispersed 0.05–0.25 mm sediment particles; *Der* (> 0.25 mm) is the difference in the percentages of effective to dispersed > 0.25 mm sediment particles.

The differences between the percentages of effective and dispersed size class aggregates in the sediments were expressed as *Der* values, which were considerably affected by the interaction between flow velocity and runoff depth (Fig 4). The effect of runoff depth on the *Der* values of the 0.05–0.25 mm particles (i.e., transport amount of microaggregates) decreased with increasing flow velocity. When the flow velocity was low, the *Der* of the 0.05–0.25 mm particles initially increased then decreased with increasing of runoff depth. The interaction effect of flow velocity and runoff depth on the *Der* values of the >0.25 mm sediment particles (i.e., percentage of macroaggregates) was smaller than that of the microaggregates. Although the *Der* values of the >0.25 mm sediment particles were slightly affected by runoff depth, the effect of runoff depth on the *Der* values of the >0.25 mm sediment particles decreased with flow velocity. The effect of flow velocity on the *Der* values of the >0.25 mm sediment particles was consistently large despite showing slight changes with runoff depth and decreased with increasing flow velocity.

### 3.3 Interaction effect of hydraulic factors on uneven OC enrichment in sediments

**Fig 5:**
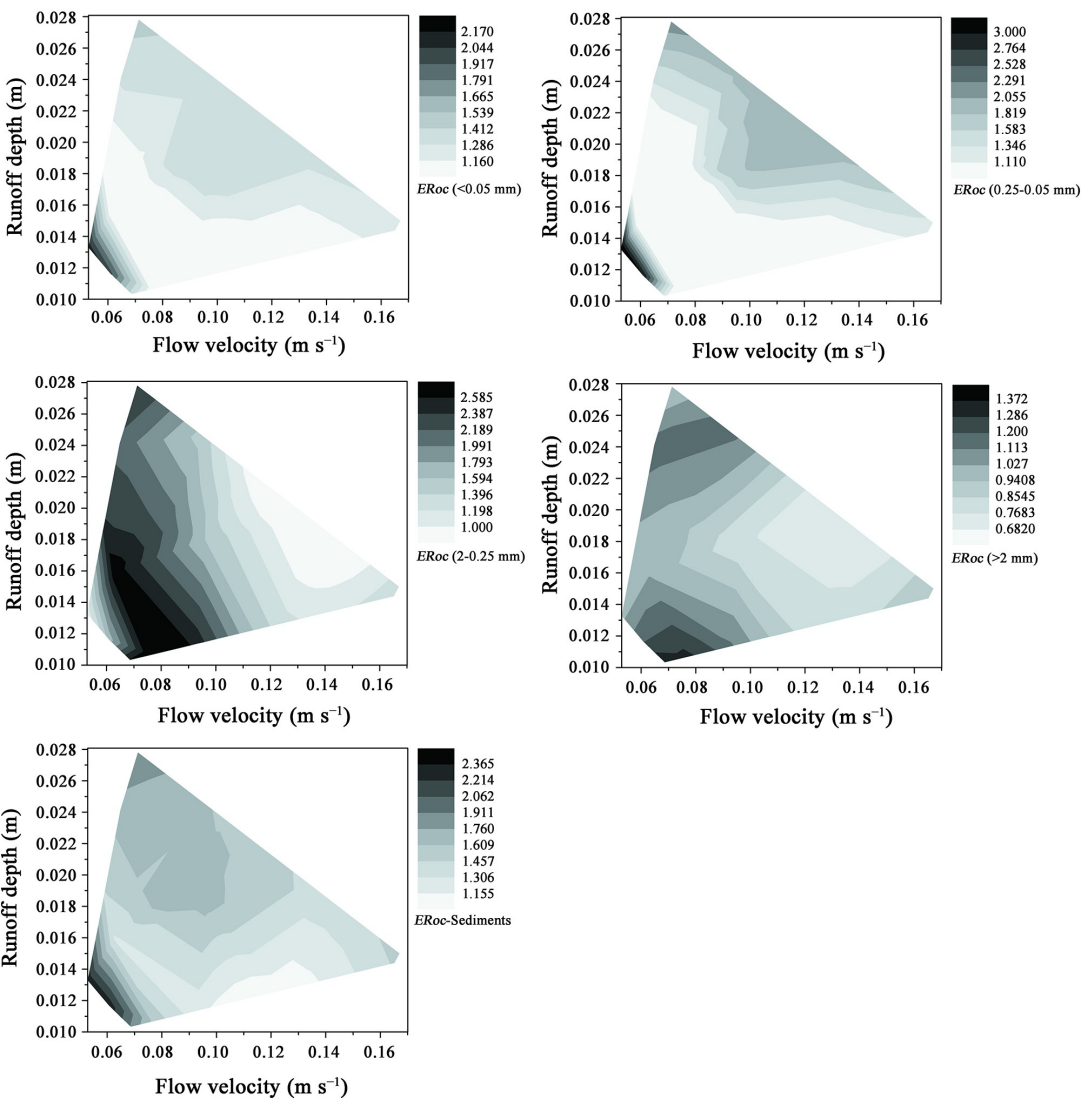
Effects of runoff depth–flow velocity interaction on *ER*_*OC*_ (OC enrichment ratios) of silt with clay-sized particles (*ER*_*oc*_ (<0.05 mm)), coarse silt (*ER*_*oc*_ (0.25–0.05 mm)), fine sand (*ER*_*oc*_ (2–0.25 mm)), coarse sand (*ER*_*oc*_ (>2 mm)), and total *ERocs* in sediment particles.

Given that flow velocity and runoff depth are the two main factors that affect uneven OC enrichment in sediment particles of different size classes [11], the interaction effects of flow velocity and runoff depth on the *ER*_*oc*_ values of sediment particles of different size classes were further investigated (Fig 5). The *ER*_*oc*_ values in silt, clay, and 0.05–0.25 mm sediment particles initially decreased rapidly with increasing runoff depth and flow velocity. When the runoff depth were enough large, the ERocs in silt, clay, and 0.05–0.25 mm sediment particles increased with the increasing of runoff depth; the critical value of runoff depth changed with the flow velocity. Thus, runoff depth and flow velocity exerted obvious interaction effects on the *ER*_*oc*_ values of silt, clay, and 0.05–0.25 mm sediment particles, especially when they were low. However, the effect of the interaction between flow velocity and runoff depth on the *ER*_*oc*_ values of silt with clay and 0.05–0.25 mm sediment particles was relatively weak when the flow velocity and runoff depth were low. The *ER*_*oc*_ values of the 0.25–2 mm sediment particles initially increased then decreased with flow velocity, and runoff depth had a smaller effect on the *ER*_*oc*_ values of the 0.25–2 mm sediment particles than flow velocity. With increasing flow velocity, the effect of runoff depth on the *ER*_*oc*_ values of the 0.25–2 mm sediment particles decreased. The *ER*_*oc*_ values of the >2 mm sediment particles generally decreased with flow velocity and initially decreased then increased with runoff depth. However, the effect of runoff depth on the *ER*_*oc*_ values of the >2 mm sediment particles decreased with increasing flow velocity. Thus, only the flow velocity was small, the *ERocs* values of 0.25–2 mm and > 2mm sediment particles have more possibilities larger than 1. Lastly, when the flow velocity was lower than 0.08 m s^−1^ and runoff depth was smaller than 0.00018 m, the total *ER*_*oc*_ values of the sediments decreased with increasing runoff depth and flow velocity. The changes in the observed trends were similar to those of *ER*_*oc*_ values in silt, clay, and 0.05–0.25 mm sediment particles. The total *ER*_*oc*_ values in the sediments were more seriously affected by runoff depth and flow velocity than those in silt, clay, and 0.05–0.25 mm sediment particles. When the flow velocity was lower than 0.8 m s^−1^, the total *ER*_*oc*_ values in the sediments were obviously larger than 1.0 even when the runoff depth was larger than 0.00018 m. This finding illustrates that sufficiently both large or small runoff depths can promote the transport of high-OC-concentration particles. All preferential transport of clay, silt and sand size particles with high OC concentrations contributed a lot to the high ERocs in the sediments.

**Fig 6:**
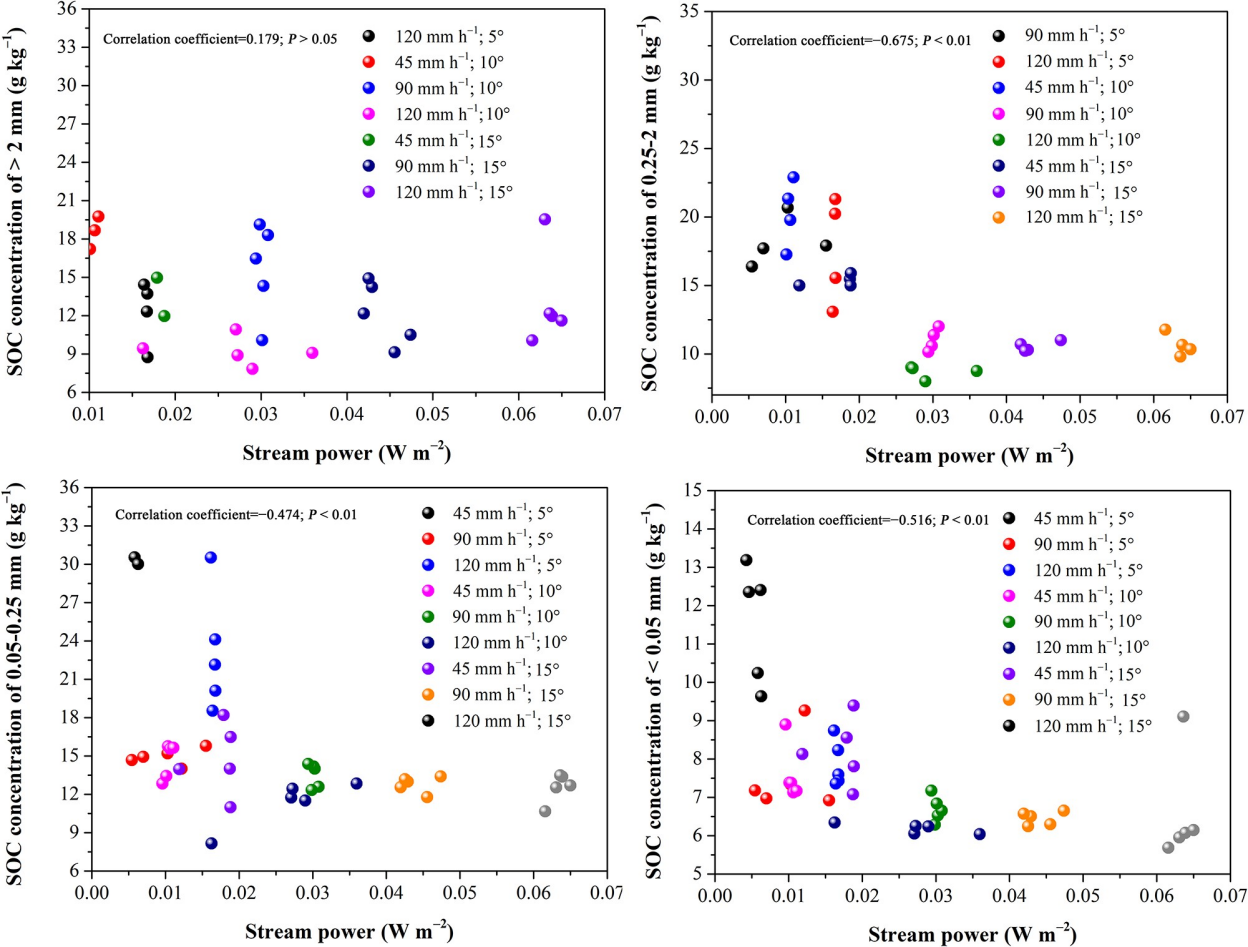
The effect of stream power on the SOC concentration of all size class sediment particles.

To investigate the effect of stream power on uneven OC enrichment in the sediments, the relations between stream power and SOC concentration in each size class of sediments are presented in Fig 6. Stream power was significantly positively correlated with OC concentration in the <0.05 mm sediment particles (*P* < 0.01), but the correlation weakened with the increase in the volume of sediment particles. Moreover, the change in the relations between stream power and OC concentration in the <0.05 and 0.25–2 mm size classes of sediment particles was more regular than that in the other particle size classes. This result may be related to the preferred transport of <0.05 mm mineral-bonded SOC and <0.05 and 0.25–2 mm free light SOC. For this loess soil, stream power had a relative small effect on OC concentration in the >2 mm and 0.05–0.025 mm size classes of particles.

### 3.4 Relationships of hydraulic factors and the contribution of uneven OC enrichment in sediments to total ERoc (*Ca*) and that of selective transport of mineral particles to total ERocs (*Sa*)

**Fig 7:**
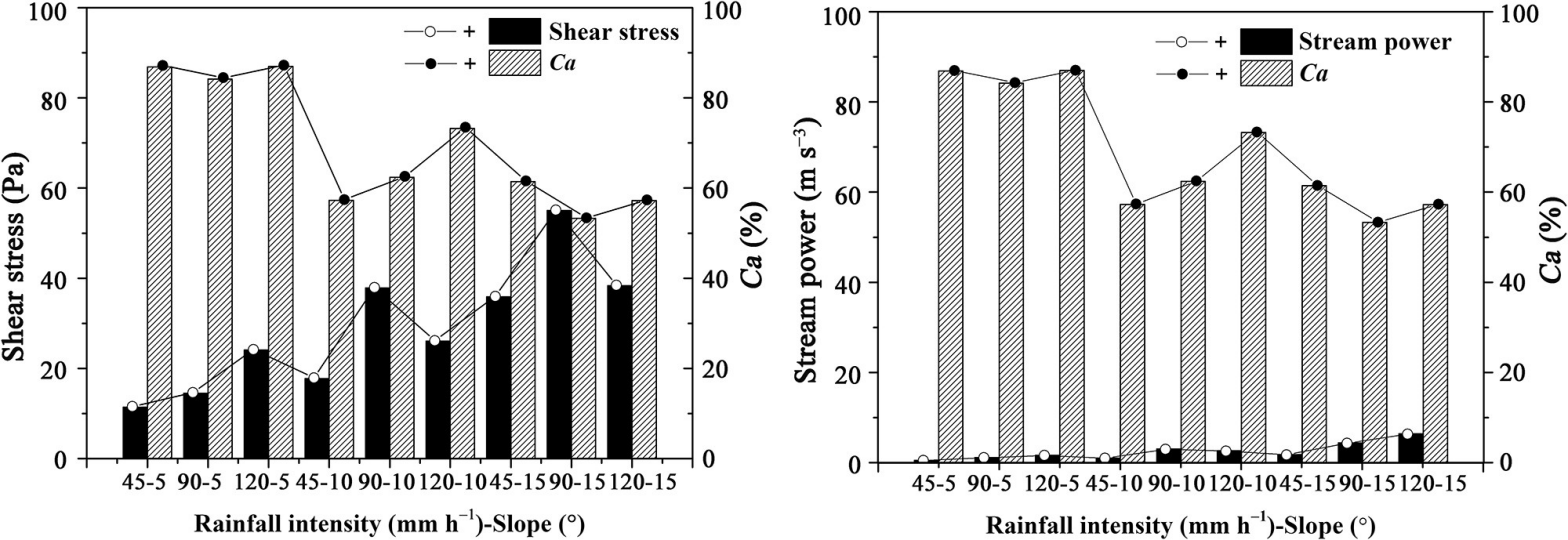
Relationships of shear stress, stream power and the contributions of unevenly enriched OC in sediments to the SOC enrichment ratios in sediments (*Ca*).

Relationships of hydraulic factors and the contributions of uneven OC enrichment in the sediments to total *ER*_*oc*_ (*Ca*) were investigated here (Fig 7). The *Ca* values decreased with increasing slope and changed less extensively with rainfall intensity than with slope. *S*hear stress, and stream power increased with slope but changed less extensively with rainfall intensity than with slope. *Ca* decreased with increasing stream power and shear stress. Analysis of the relationships between the total *ER*_*oc*_ values in the sediments and hydraulic factors under different rainfall conditions (Tables 2 and 3) revealed that the former showed the same change trends as the *Ca* values. Thus, increments in slope weakened SOC enrichment in sediments more than increments in rainfall intensity did because of the effects of runoff hydraulic characteristics, such as shear stress and stream power, on uneven OC enrichment in the sediments. However, the subtle effect of rainfall intensity on the runoff hydraulic characteristics and even *Ca*, was complex. According to the results of Pearson correlation analysis, *Ca* was significantly correlated with shear stress (*P* < 0.05) but not with flow velocity, runoff depth, or stream power (Table 4). The total *ER*_*oc*_ values in the sediments were significantly correlated with shear stress (*P* < 0.05) and stream power (*P* < 0.01) but not with flow velocity or runoff depth. Therefore, the interaction between slope, runoff depth and flow velocity greatly affected *Ca* and the uneven *ER*_*oc*_ values of different size classes of sediment particles.

**Table 4:** Pearson correlation analysis results of flow hydraulic characteristics, contribution of unevenly enriched orgainc carbon in sediments to *ERoc* (*Ca*) and *ERocs*.

Coorelation cofficients	Flow velocity (m s^-1^)	Runoff depth (m)	Shear stress (Pa)	Stream power (g s^-3^)	*Ca* (%)	*ERoc*
Flow velocity (m s^-1^)	1	-0.058	0.335	0.854**	-0.336	-0.510
Runoff depth (m		1	0.316	0.128	0.265	-0.324
Shear stress (Pa)			1	0.760*	-0.736*	-0.846**
Stream power (g s^-3^)				1	-0.614	-0.759*
*Ca* (%)					1	0.702*
*ERoc*						1

### 3.5 Quantitative relationships between hydraulic factors and uneven *ERoc* values in the sediments

**Fig 8:**
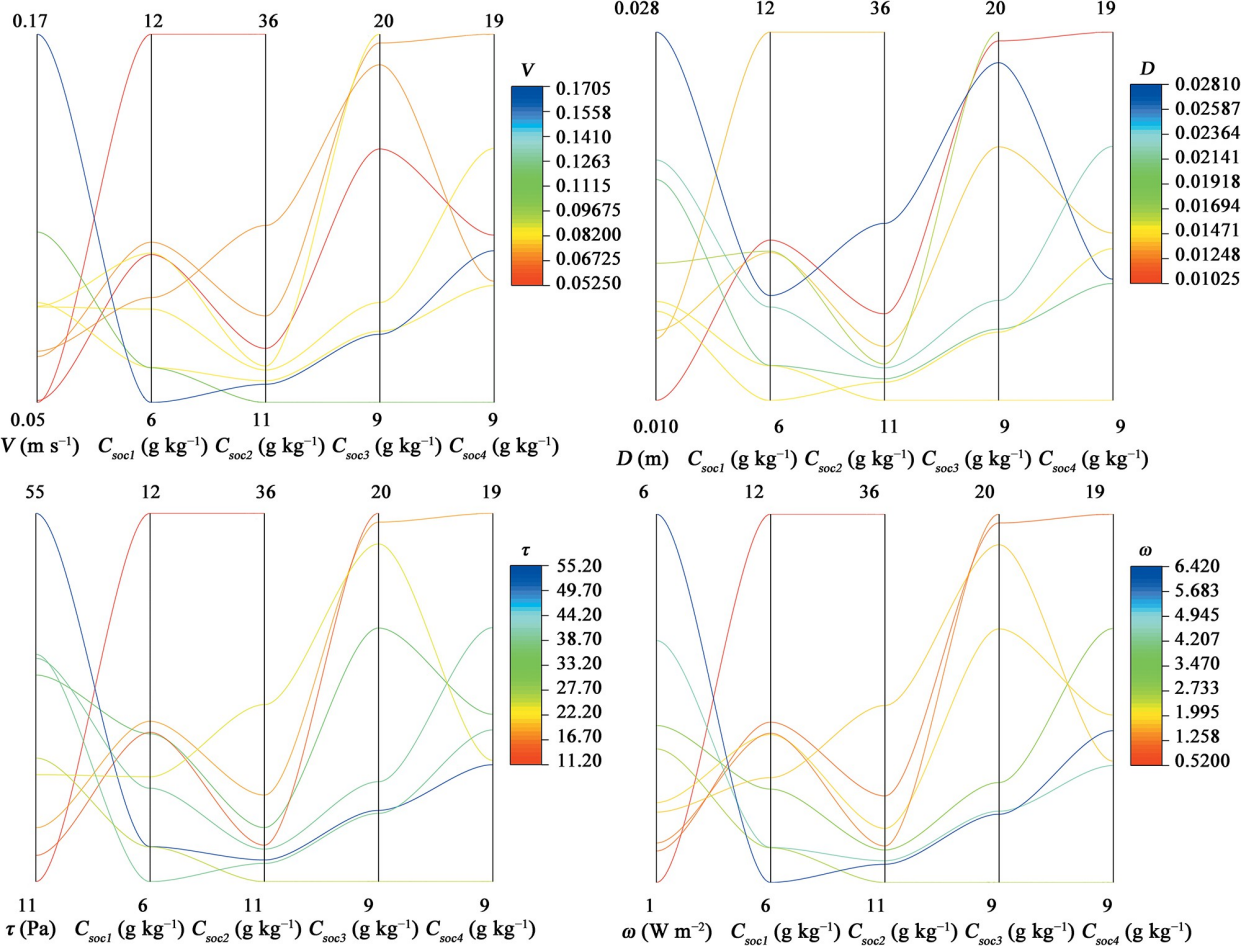
Effect of hydraulic characteristics (e.g., flow velocity, runoff depth, shear stress, and stream power) on OC distribution in sediments (*C*_*soc1*_: The SOC concentration in < 0.05 mm sediment particles; *C*_*soc2*_: The SOC concentration in 0.05–0.25 mm sediment particles; *C*_*soc3*_: The SOC concentration in 0.25–2 mm sediment particles; *C*_*soc4*_: The SOC concentration in > 2 mm sediment particles).

Given the interaction effects of runoff hydraulic characteristics on uneven OC enrichment in different size classes of sediment particles and the effect of aggregate transport during soil erosion, the internal relationships of OC concentration in the different size classes of sediment particles and four main hydraulic factors, namely, flow velocity, runoff depth, shear stress, and stream power, were further investigated (Fig 8). Flow velocity was negatively correlated with OC concentration in the different size classes of sediment particles, but these relationships are different for different size classes of sediment particles. When the runoff depth was sufficiently small, the OC concentrations in the <0.25 mm sediment particles were consistently high. When the runoff depth was sufficiently large, the OC concentrations in the >0.25 mm sediment particles were consistently high. The OC concentration in silt and clay obviously decreased with flow velocity and runoff depth. Compared with flow velocity and runoff depth, shear stress and stream power showed a closer relationship with OC concentration in the sediment particles in each size class. However, due to the impact of rain and other direct or interacting factors, such as critical slope for soil erosion, aggregate content, slope, and rainfall intensity may be more suitable for OC enrichment prediction than the product of slope and runoff depth or flow velocity, namely, shear stress or stream power. The soil aggregate instability index was incorporated into the inter-rill soil erosion rate equation because it influences soil erodibility and the size distributions of the products of aggregate breakdown [42–44] The destruction of aggregates is closely related to the distance of aggregate transport [45]. Aggregate stability has a considerable effect on SOC enrichment in sediments [46]. In this study, the effective median diameters of the sediments (*D*_*50*_), flow velocity, rainfall intensity, and slope were incorporated into the following nonlinear regression equations to present the changes in OC distribution in the sediments. The equations were shown as follows:

$$C_{oc-distribution}=C_{soc^{\prime }}\times \rho_{s}\times R^{a}\times S^{b}\times {\left(V\times D\right)}^{c}\times D_{50}^{d},$$ (8)

$$C_{oc-distribution}=C_{soc^{\prime }}\times \rho_{s}\times R^{a}\times S^{b}\times V^{c}\times D^{d},$$ (9)

where *C*_*oc-distribution*_ is the OC concentration of sediment aggregates in the different size classes (g kg^−1^), *C*_*soc’*_ is the original SOC concentration in different size classes of sediment particles, *ρ*_*s*_ is the material density (kg m^−3^), *R* is the rainfall intensity (mm h^−1^), *S* is the slope (m m^−1^), *V* is the flow velocity (m s^−1^), *D* is the runoff depth (m), *D*_*50*_ is the median diameter of the sediment particles, and *a*, *b*, *c*, and *d* are correlation coefficients that are mainly related to the properties of the original soil. The OC concentrations in clay with <2 mm sediment particles were calculated using Equation (4). The OC concentrations in the >2 mm sediment particles were calculated using Equation (5). The regression coefficients of the two functions for OC concentrations in the sediment particles in the four size classes are shown in Table 5. According to the regression coefficients obtained, flow velocity, runoff depth, *D*_*50*_, rainfall intensity, and slope were negatively correlated with OC concentrations in the <0.05 mm silt with clay sediment particles. Large values of rainfall intensity and slope weakened the uneven OC enrichment in the different size classes of sediments. Rainfall intensity and slope presented interactions with flow velocity and runoff depth in OC concentration in the 0.05–0.25 and 0.25–2 mm sediment particles, especially the former. This result is consistent with our mechanism analysis. Thus, to some degree, the function can represent the effects of flow velocity, runoff depth, rainfall intensity, and slope on the OC concentrations of sediment particles in each size class. The *d* values were negative for the OC concentrations of sediments in the three size classes, indicating that high aggregate stability and coarse sediment size distribution may have decreased the *ER*_*oc*_ values of the different size classes of sediment particles. Therefore, the OC concentrations in each size class of sediments were more greatly affected by direct factors, such as rainfall intensity, than by hydraulic factors; however, the internal effects of hydraulic factors were also important. In addition, the average calculated errors of OC concentrations in the sediment particles of different size classes were below 0.30. The regression accuracy is shown in Table 6. The *R*^*2*^ values of the function for OC concentrations in the <0.05, 0.05–0.25, 0.25–2, and >2 mm sediment particles were 0.965, 0.994, 0.844, and 0.805, respectively.

**Table 5:** *a*, *b*, *c*, and *d* values for the OC uneven enrichment in different size class sediments prediction function.

Parameters	< 0.05 mm	0.05–0.25 mm	0.25–2 mm	> 2 mm
*a*	−0.035±0.189	1.804±0.175	0.120±0.487	−0.868±0.070
*b*	−0.093±0.133	0.893±0.128	−0.254±0.314	−0.313±0.167
*c*	−0.054±0.018	−0.099±0.015	−0.136±0.053	0.605±0.136
*d*	−0.382±0.241	−2.874±0.223	−0.831±0.690	0.283±0.146

**Table 6:** Calculated results of the OC distribution in sediments regressed function.

Treatment (rainfall intensity-slope)	C_SOC_-calculated (< 0.05 mm; g kg^−1^)		C_SOC_-calculated (0.05–0.25 mm; g kg^−1^)		C_SOC_-calculated (0.25–2 mm; g kg^−1^)		C_SOC_-calculated (> 2 mm; g kg^−1^)	
	Values	Errors	Values	Errors	Values	Errors	Values	Errors
45–5	11.27	0.30	35.80	−0.02	none	none	none	none
45–10	8.86	−0.62	17.63	−0.54	20.51	−0.74	17.74	0.81
45–15	7.86	0.19	13.73	1.21	15.48	1.05	14.72	−1.25
90–5	7.99	0.08	14.90	−1.13	19.14	0.90	none	none
90–10	6.77	0.41	14.18	−0.68	12.83	−1.01	13.69	1.97
90–15	6.25	−0.01	13.53	−0.74	10.58	0.36	12.02	0.18
120–5	7.77	−0.41	22.10	0.99	18.97	0.13	12.70	−0.39
120–10	6.21	0.04	10.69	0.65	11.27	−2.51	11.17	−1.93
120–15	5.64	0.05	12.46	0.09	8.88	1.97	12.90	0.18
Average	11.27	0.30	35.80	−0.02	20.51	−0.74	13.56	0.79
Coefficients of determination	*R*^*2*^ = 0.965		*R*^*2*^ = 0.994		*R*^*2*^ = 0.844		*R*^*2*^ = 0.805	

**Fig 9:**
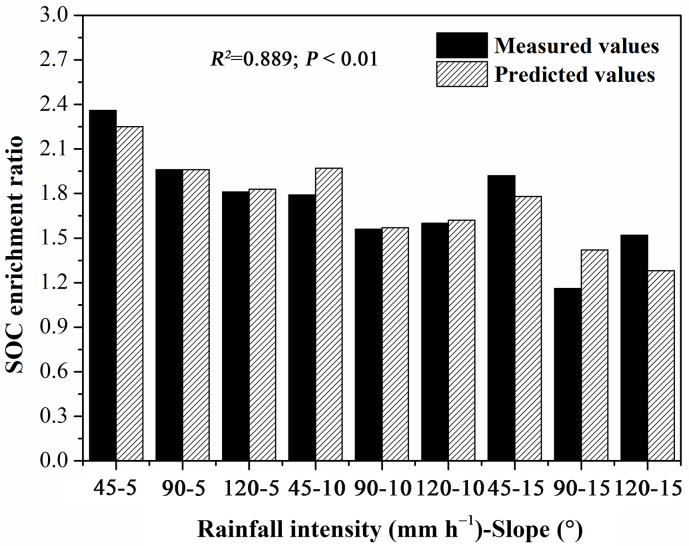
Calculated results of *ERocs* regressed equation.

According to the interaction effects of flow velocity, runoff depth, and slope on uneven OC enrichment in the different size classes of sediment particles and the close relationships among shear stress, stream power, *Ca*, and *ER*_*oc*_, the total *ER*_*oc*_ values in the sediments logarithmically decreased with flow velocity, runoff depth, and slope as follows:

$$ERocs=k\times \text{ln}(S\times D\times V)+e\;\left(R^{2}=0.789\;\text{and}\;P<0.005\right),$$ (10)

where *S* is the slope (m m^−1^), *V* is the flow velocity (m s^−1^), *D* is the runoff depth (m), and *k* and *e* are correlation coefficients that are mainly related to the properties of the original soil or sediment particle size distribution. The regression coefficients of the equation are shown in Table 7. According to regression coefficient *k*, the *ER*_*oc*_ values were negatively correlated with the product of slope, flow velocity, and runoff depth. These findings are consistent with the effects of flow velocity, runoff depth, shear stress, stream power, and slope on the OC concentration in the sediments. The absolute average calculated errors of the *ER*_*oc*_ values in the sediments were below 0.26 (Table 8), and the *R*^*2*^ values of the equation for the *ER*_*oc*_ values in the sediments were 0.789 (Fig 9).

**Table 7:** Regression results of the *ERoc* calculated equation.

correlation coefficients	values	SD
*a*	-0.409	0.080
*b*	-0.065	0.357

**Table 8:** Measured and calculated *ERocs* under different rainfall intensities and slopes.

Treatments	Rainfall intensity (mm h^-1^)-Slope (°)								
	45–5	90–5	120–5	45–10	90–10	120–10	45–15	90–15	120–15
Measured *ERocs*	2.36	1.96	1.81	1.79	1.56	1.6	1.92	1.16	1.52
Calculated *ERocs*	2.25	1.96	1.83	1.97	1.57	1.62	1.78	1.42	1.28
RESID	0.12	0	-0.02	-0.18	-0.01	-0.03	0.14	-0.26	0.24

## 4. Discussion

### 4.1 Hydraulic mechanisms of unevenly enriched OC in sediments

The changing trend of different hydraulic characteristics was due to the decrease of slope runoff infiltration rate with time. Therefore, the runoff rate and runoff depth increased with time when the rainfall intensity was larger or equal to 90 and 120 mmh^−1^. However, owing to the increasing detached difficulty of large soil particles, the flow velocity decreased with time when the rainfall intensity was large. The shear stress also decreased with time under large rainfall intensity. To the contrary, the stream power increased and quickly became stable with time, corresponding with the sediment loss rate. In this study, the high flow velocities have a positive exponential correlation with the transport number of >0.05 mm aggregates with high density and low OC concentration [16, 17]. This is because the erosion is controlled by transport limitation. At such situation, small and large runoff depths accompanied with small velocity can result in high uneven *ER*_*oc*_ values in different size classes of sediment particles. Flow velocities in the range of approximately 0–0.08 m s^−1^ cause obvious uneven OC enrichment in the sediments. Large runoff depths and low flow velocities cause obvious enrichment of OC in > 0.25 large particles, while small runoff depths and low flow velocities mainly promote the build-up of high OC concentrations in clay and silt. Under the low flow velocity and stream power generated by high runoffs caused by high rainfall intensities and small slopes, the small original grain particles (<0.02 mm), including clay, silt, and high-OC-concentration light large particles, are mainly preferentially washed down, most of which is supplied by new materials being fragmented by raindrops [47]. At this time, the OC mineralization potential of the sediments that can be deposited in low location may be large [48, 49]. Under high runoff erosive power, that is, high flow velocity and stream power, the high sediment erosion rate (*Qs*) weakens the effect of aggregate stripping on uneven OC enrichment in sediments [16]. The OC mineralization potential of deposited sediments may be low because of the high soil erosion rate. Thus, these studies provide an important reference and possibility for evaluating SOC mineralization rates in details.

Comparing the hydraulic mechanisms of OC enrichment between different size class particles shows that the critical flow velocity of the transport of large light OC-enriched particles is relative larger than that of the clay and silt with high OC concentration. The ranges of flow velocity and runoff depth that contribute considerably to the high *ER*_*ocs*_ in > 0.25 mm size class particles are larger than those in < 0.25 mm size class particles. This finding is associated with the transport style and force situation of different particles in runoff [17, 50]. During erosion, the values of critical flow velocity and stream power for the transport of organic/inorganic soil particles with different sizes and densities determined the transport order and amounts of these particles. For example, the order of soil particles according to their critical velocity was clay, silt, large size and light particulate OC, small to large aggregate fragments, and sand. Clay and silt were preferred to be transported first. Large size and light particulate OCs were next, part of which were produced through aggregate stripping determined by rainfall characteristics. Small to large aggregate fragments and sand followed. Our study shows that the interaction between runoff depth and flow velocity for the transport of high- or low-OC-concentration small aggregate fragments is more enhanced when the sediment particle size is smaller. The transport limit hydraulic erosion situation contributes considerably to the great effect of flow velocity on sediment size distribution and OC. With the increase in runoff erosive power, an abundant amount of large heavy particles are transported, and the flow velocity is positively correlated with the amounts of heavy aggregates in sediments.

### 4.2 Roles of slope, rainfall intensity, and hydraulic factors for predicting uneven OC enrichment in different size particles

Among hydraulic parameters, stream power is most significantly correlated the uneven enrichment of SOC in the sediments, which is a product of slope and flow velocity. Except runoff hydraulic features, slope is an essential factor for predictions of soil erosion and uneven OC enrichment in the sediments. Therefore, the combined effects of runoff depth, flow velocity, and slope determine the ultimate contribution of raindrop peeling on the uneven *ER*_*oc*_ values in all size classes of sediment particles. In addition, aggregate hierarchy theory posits that many microaggregates are wrapped by macroaggregates [51, 52]. This aggregate hierarchy in soils explains the effect of aggregate breakdown on transport of SOC and its uneven enrichment in sediments. Aggregate stripping produces large amounts of small particles with different OC concentrations during water erosion [14, 53, 54]. The resulting light and high OC concentration particles are preferentially transported through runoff. Thus, OC is unevenly enriched in different size classes of sediment particles. In fact, soil aggregate stripping is greatly affected by raindrop intensity [17]. Vaezi et al. [55] verified that the effect of raindrop intensity on sediment transport increases with the decrease in rainfall intensity. Thus, rainfall intensity has an important single effect on the amount and size of original materials that will be selectively tranported. The effect of rainfall intensity cannot be ignored when predicting sediment size distribution and *ER*_*oc*_ distribution between different size class sediment particles by runoff hydraulic characteristics. Hydraulic factors mainly determine the OC enrichment features in sediments by affecting the following selective transport process of free organic matter, mineral particles, and aggregate fractions. These findings may not be generalizable to other soils with low SOC and aggregate contents due to the high SOC concentration and aggregate content in loess soil. They may also not be generalizable to detachment limit erosion situation. These deductions should be verified in future research.

### 4.3 Relational regression functions of hydraulic factors and uneven OC enrichment in different size classes of sediments

In the WEPP model, the inter-rill soil erosion rate has been estimated using the formula that relates the inter-rill erodibility coefficients with slope, rainfall intensity, and hydraulic factors [56]. However, the single or interaction effect of slope, rainfall intensity and hydraulic factors between soil erosion rate and SOC enrichment in sediments is different. Furthermore, aggregate stability has a considerable effect on soil erosion and SOC enrichment in sediments [46]. Hence, *D*_*50*,_ slope, rainfall intensity and hydraulic factors should be incorporated into the SOC enrichment prediction functions. For the choosing of hydraulic factors, although shear stress and stream power have a closer relationship with *ER*_*oc*_ values than flow velocity and runoff depth, we considered the subtle interactions among the effects of slope, runoff depth, and flow velocity because they are important in determining uneven OC enrichment in different size classes of sediments. Thus, runoff depth, and flow velocity were incorporated into our regression functions. These functions yielded good fitting results (*R*^*2*^ > 0.789; *P* < 0.005). This finding further illustrates that hydrology is essential to SOC loss models and improves prediction accuracy [57, 58]. However, whether the functions can be used to predict SOC transport and *ER*_*oc-i*_ that is closely related to OC mineralization should be verified. Our study further demonstrated the possibility of SOC enrichment prediction in details. In the future, this approach should be further investigated by tracking the changes in SOC labile factions in aggregates affected by sediment erosion and deposition.

According to the parameters in regression functions, the SOC transport regression equations in our study can describe the OC concentrations in different sediment size classes and reveal the mechanisms of hydraulic factors in SOC transport during erosion to some degree. The effect of rainfall intensity and slope on the *ER*_*ocs*_ of sediment size classes became large with increasing sediment particle size. Flow velocity and runoff depth exerted more obvious effects on OC concentrations in small sediment particles than that in other sediment size classes, and *D*_*50*_ had large effect on OC concentrations in the 0.05–0.25 mm sediment particles. Furthermore, the individual effects of flow velocity and runoff depth played a more important role in OC enrichment in the >0.25 mm sediment particles than in their interaction effects. However, the interaction effects of flow velocity and runoff depth determined the OC enrichment features of silt and clay that mainly determined the total *ER*_*oc*_ values in the sediments. Therefore, the exponential functions that incorporates the product of flow velocity, runoff depth, and slope as an independent variable may be used to predict uneven SOC enrichment in different size classes of sediments. Given that the prediction of SOC loss induced by water erosion can be roughly calculated by current SOC models [8, 23], our proposed function can provide an important reference for improving SOC models, such as the CENTURY model.

## 5. Conclusions

This study investigates the hydraulic transport mechanisms of uneven OC enrichment in sediments and the equations representing the relationships between uneven *ER*_*oc*_ values in different size classes of sediment particles, erosion conditions, and runoff hydraulic factors. The single and interaction effect of flow velocity, runoff depth, rainfall intensity, and slope determine the uneven OC enrichment in each size class of sediment particles. From our study, stream power and shear stress are greatly positively correlated with the OC concentration in different size classes of sediments in different ways. However, flow velocity and runoff depth, as runoff hydraulic parameters, can better explain the OC enrichment mechanisms in the sediments than shear stress and stream power due to their great single effect. Slope, rainfall intensity, and aggregate stability also cannot be ignored because the first one is greatly interacted with runoff hydraulic characteristics and the following two represent the aggregate breakdown feathers. These factors all greatly affect the uneven enrichment of OC between sediment particles, and they cannot be substituted by runoff hydraulic factors. Hydraulic factors mainly affect the selective transport of organic/inorganic soil particles with different sizes and densities. The interaction of flow velocity and runoff depth on preferred transport of light particles enhances with the decrease in particle size. The individual effects of flow velocity and runoff depth play a more important role in OC enrichment in the >0.25 mm sediment particles than in their interaction effects. The high-OC-concentration particles with clay and silt sizes are easier to be transported than the high-OC-concentration particles with a large size due to the different of critical flow velocities of organic/inorganic soil particles with different sizes and densities. Furthermore, the uneven OC enrichment in the different size classes of particles was not associated with the transport of heavy aggregates needing large runoff erosive power.

According to the hydraulic mechanisms and effect factors of OC uneven enrichment between different size sediment particles, relational regression functions of uneven OC enrichment in different size classes of sediment particles, flow velocity, slope, runoff depth, sediment median diameter, and rainfall intensity were built. The *ER*_*oc*_ regression functions of the different size classes of particles differed between large and small particle size classes. To some degree, the regression coefficients could present the effect of associated input factors and the interaction effects of slope and hydraulic factors. Our study about uneven SOC enrichment in different size classes of sediment particles could provide an important reference for evaluating OC mineralization under water erosion and further investigating SOC turnover under water erosion.

## Supporting information

S1 Dataset (DOCX)
