# Supplementary material for: Hydraulic mechanisms of the uneven enrichment of soil organic carbon in sediments during rain-induced overland flow
Source: PLoS One. 2022 Feb 22;17(2):e0262865. doi: 10.1371/journal.pone.0262865 (PMC8863236; doi:10.1371/journal.pone.0262865)
Supplement: S1 Dataset — (DOCX) [file pone.0262865.s001.docx]

**The Data of Figure 2**

| Rainfall intensity  (mm h^−1^)-Slope (°) | 45-5 | | 90-5 | | 120-5 | |  | 45-10 | | 90-10 | | 120-10 | |  | 45-15 | | 90-15 | | 120-15 | |
| --- | --- | --- | --- | --- | --- | --- | --- | --- | --- | --- | --- | --- | --- | --- | --- | --- | --- | --- | --- | --- |
| Time (*min*) | *V* | STDVE | *V* | STDVE | *V* | STDVE | Time (*min)* | *V* | STDVE | *V* | STDVE | *V* | STDVE | Time (*min*) | *V* | STDVE | V | STDVE | V | STDVE |
| 3 | 0.0642 | 0.00827 | 0.06933 | 0.01359 | 0.0653 | 0.00357 | 3 | 0.03542 | 0.00386 | 0.11655 | 0 | 0.10214 | 0.01254 | 3 | 0.06232 | 0.00901 | 0.14347 | 0.01526 | 0.19255 | 0.02432 |
| 6 | 0.03508 | 1.60E-04 | 8.89E-02 | 0.0015 | 0.07213 | 0.00363 | 6 | 0.04832 | 0.00902 | 0.08597 | 0.01423 | 0.11235 | 0.01889 | 6 | 0.03919 | 0.00248 | 0.1202 | 0.01103 | 0.21088 | 0.02721 |
| 9 | 0.02737 | 0.00279 | 0.06332 | 0.00471 | 0.08315 | 0.01567 | 9 | 0.0419 | 0.00492 | 0.09945 | 0.00582 | 0.11356 | 0.02809 | 9 | 0.04172 | 5.22E-05 | 0.11038 | 7.31E-04 | 0.17676 | 0.02105 |
| 12 | 0.03874 | 0.00425 | 0.11504 | 0.02462 | 0.07764 | 3.62E-04 | 12 | 0.06544 | 0.0015 | 0.08554 | 0.00862 | 0.1055 | 0.00134 | 12 | 0.04952 | 0.01021 | 0.11529 | 0.01003 | 0.17343 | 0.01214 |
| 15 | 0.02979 | 7.54E-04 | 7.40E-02 | 0.00599 | 0.06924 | 0.00698 | 15 | 0.05335 | 0.01561 | 0.10441 | 0.00381 | 0.09281 | 0.0066 | 15 | 0.07757 | 0.01204 | 0.08266 | 0.00266 | 0.17244 | 0.01086 |
| 18 | 0.0279 | 0.00119 | 0.10443 | 0.0289 | 0.06698 | 5.83E-04 | 18 | 0.04459 | 9.93E-04 | 0.07247 | 0.00553 | 0.0915 | 0.00109 | 18 | 0.08227 | 0.02762 | 0.09675 | 0.02007 | 0.15752 | 0.01489 |
| 21 | 0.03066 | 1.13E-04 | 8.90E-02 | 0.02337 | 0.07748 | 0.00656 | 21 | 0.07424 | 1.65E-04 | 0.09175 | 0.024 | 0.10138 | 0.01047 | 21 | 0.05096 | 0.00957 | 0.08519 | 0.00827 | 0.19544 | 0.01289 |
| 24 | 0.03373 | 0.00114 | 0.11124 | 0.03582 | 0.06297 | 0.00415 | 24 | 0.06065 | 0.02631 | 0.09056 | 0.0036 | 0.10324 | 0.01991 | 24 | 0.05508 | 0.01514 | 0.07521 | 0.00854 | 0.18878 | 0.00435 |
| 27 | 0.03381 | 0.00323 | 0.09309 | 0.01802 | 0.07304 | 0.01243 | 27 | 0.03783 | 0.00299 | 0.08591 | 0.00243 | 0.11329 | 0.00576 | 27 | 0.0527 | 0.01626 | 0.08368 | 3.50E-04 | 0.16308 | 0.00737 |
| 30 | 0.03189 | 0.00204 | 0.07743 | 0.01014 | 0.06714 | 0.00839 | 30 | 0.08962 | 0.00128 | 0.0895 | 0.00574 | 0.09992 | 0.02056 | 30 | 0.06214 | 0.0048 | 0.08395 | 0.01313 | 0.17342 | 0.001 |
| 33 | 0.04448 | 0.01028 | 0.08166 | 0.01543 | 0.06383 | 0.00669 | 33 | 0.04998 | 0.01927 | 0.07882 | 0.00859 | 0.118 | 0.01899 | 33 | 0.04845 | 0.00754 | 0.06117 | 5.61E-04 | 0.17491 | 0.00509 |
| 36 | 0.05323 | 0.01913 | 0.07383 | 0.01514 | 0.07248 | 0.00445 | 36 | 0.06028 | 0.00765 | 0.088 | 0.0067 | 0.14237 | 0.01148 | 36 | 0.03434 | 0.00305 | 0.08127 | 0.00435 | 0.18546 | 0.01724 |
| 39 | 0.06861 | 0.01013 | 0.08572 | 0.00636 | 0.06796 | 0.00217 | 39 | 0.10439 | 0.01697 | 0.08399 | 0.00562 | 0.11296 | 0.01492 | 39 | 0.04988 | 1.74E-04 | 0.06558 | 8.17E-04 | 0.16087 | 0.00158 |
| 42 | 0.06747 | 0.00694 | 0.07535 | 0.00868 | 0.06927 | 0.00278 | 42 | 0.06922 | 0.00817 | 0.07948 | 0.01013 | 0.10373 | 0.00664 | 42 | 0.05064 | 0.00205 | 0.07239 | 0.01322 | 0.14348 | 0.00286 |
| 45 | 0.06758 | 0.00562 | 0.06173 | 3.81E-04 | 6.16E-02 | 0.00378 | 45 | 0.04896 | 0.00952 | 0.05765 | 0.00858 | 0.10145 | 0.03296 | 45 | 0.0588 | 0.01293 | 0.06157 | 0.00212 | 0.15781 | 0.02041 |
| 48 | 0.08159 | 0.01016 | 0.09705 | 0.01977 | 0.06432 | 0.00371 | 48 | 0.10491 | 0.02199 | 0.07582 | 7.47E-04 | 0.12726 | 0.022 | 48 | 0.05437 | 0.00587 | 0.06992 | 0.00515 | 0.16762 | 0.00546 |
| 51 | 0.0875 | 0.00458 | 0.0666 | 0.00124 | 0.06958 | 0.00629 | 51 | 0.06474 | 0.00955 | 0.07616 | 0.01592 | 0.10413 | 0.02745 | 51 | 0.0451 | 0.00535 | 0.07343 | 0.00531 | 0.16764 | 0.03371 |
| 54 | 0.07434 | 0.00441 | 0.0798 | 0.01366 | 0.06311 | 9.95E-04 | 54 | 0.11426 | 0.02235 | 0.06985 | 0.00423 | 0.09708 | 0.01708 | 54 | 0.05227 | 0.00391 | 0.07916 | 0.01276 | 0.15419 | 0.00567 |
| 57 | 0.08501 | 0.00844 | 0.07237 | 0.00397 | 0.07144 | 0.00548 | 57 | 0.09257 | 0.00248 | 0.07233 | 0.0024 | 0.11028 | 0.00654 | 57 | 0.05519 | 6.09E-05 | 0.06219 | 0.00158 | 0.13206 | 0.01333 |
| 60 | 0.07442 | 0.00545 | 0.09031 | 0.01958 | 0.06492 | 0.00122 | 60 | 0.08835 | 0.00873 | 0.06133 | 9.40E-04 | 0.08847 | 0.01034 | 60 | 0.04773 | 0.00309 | 0.06904 | 0.01658 | 0.15286 | 0.00532 |

*V*: Flow velocity (m s^−1^).

| Rainfall intensity  (mm h-1)-Slope (°) | 45-5 | | 90-5 | | 120-5 | |  | 45-10 | | 90-10 | | 120-10 | |  | 45-15 | | 90-15 | | 120-15 | |
| --- | --- | --- | --- | --- | --- | --- | --- | --- | --- | --- | --- | --- | --- | --- | --- | --- | --- | --- | --- | --- |
| Time (*min*) | *D* | STDVE | *D* | STDVE | *D* | STDVE | Time (min) | *D* | STDVE | *D* | STDVE | *D* | STDVE | Time (min) | *D* | STDVE | *D* | STDVE | *D* | STDVE |
| 3 | 9.61E-05 | 4.11E-06 | 1.98E-04 | 7.03E-05 | 2.57E-04 | 7.21E-05 | 3 | 1.48E-04 | 1.76E-05 | 1.52E-04 | 4.57E-06 | 9.54E-05 | 2.59E-05 | 3 | 9.71E-05 | 1.48E-05 | 1.18E-04 | 1.26E-05 | 1.24E-04 | 1.06E-05 |
| 6 | 1.35E-04 | 1.28E-05 | 1.63E-04 | 1.56E-05 | 2.49E-04 | 2.30E-06 | 6 | 1.16E-04 | 2.85E-05 | 2.08E-04 | 4.41E-05 | 8.00E-05 | 2.51E-06 | 6 | 1.62E-04 | 3.45E-06 | 1.15E-04 | 2.73E-05 | 1.19E-04 | 2.43E-05 |
| 9 | 2.02E-04 | 3.04E-05 | 2.30E-04 | 4.88E-05 | 2.28E-04 | 2.91E-05 | 9 | 1.36E-04 | 7.47E-06 | 1.70E-04 | 2.88E-06 | 1.57E-04 | 9.79E-05 | 9 | 1.61E-04 | 4.77E-06 | 1.45E-04 | 1.72E-06 | 1.42E-04 | 6.14E-06 |
| 12 | 1.30E-04 | 1.65E-05 | 1.25E-04 | 5.29E-06 | 2.43E-04 | 1.51E-05 | 12 | 8.51E-05 | 7.59E-06 | 2.07E-04 | 2.51E-05 | 8.93E-05 | 7.75E-06 | 12 | 1.43E-04 | 2.68E-05 | 1.16E-04 | 3.84E-05 | 1.40E-04 | 2.07E-05 |
| 15 | 1.75E-04 | 3.42E-06 | 1.78E-04 | 2.64E-05 | 2.72E-04 | 1.09E-05 | 15 | 1.21E-04 | 4.25E-05 | 1.69E-04 | 1.15E-05 | 9.77E-05 | 1.94E-05 | 15 | 9.21E-05 | 1.41E-05 | 1.42E-04 | 6.36E-05 | 1.44E-04 | 2.00E-05 |
| 18 | 1.99E-04 | 1.31E-05 | 1.22E-04 | 2.00E-06 | 2.92E-04 | 1.57E-05 | 18 | 1.34E-04 | 8.38E-06 | 2.50E-04 | 2.40E-05 | 1.69E-04 | 6.70E-05 | 18 | 9.66E-05 | 3.26E-05 | 1.70E-04 | 4.43E-05 | 1.57E-04 | 2.52E-06 |
| 21 | 1.74E-04 | 2.22E-06 | 1.37E-04 | 8.85E-06 | 2.53E-04 | 8.29E-06 | 21 | 7.84E-05 | 5.16E-06 | 2.09E-04 | 5.11E-05 | 1.51E-04 | 4.74E-05 | 21 | 1.42E-04 | 2.44E-05 | 1.23E-04 | 4.97E-05 | 1.22E-04 | 1.66E-05 |
| 24 | 1.60E-04 | 5.19E-06 | 1.06E-04 | 5.76E-06 | 3.10E-04 | 2.21E-07 | 24 | 1.23E-04 | 6.10E-05 | 1.97E-04 | 3.62E-06 | 1.69E-04 | 9.05E-05 | 24 | 1.40E-04 | 3.74E-05 | 1.57E-04 | 9.52E-05 | 1.29E-04 | 1.20E-05 |
| 27 | 1.63E-04 | 1.14E-05 | 1.20E-04 | 2.58E-05 | 2.77E-04 | 2.96E-05 | 27 | 1.55E-04 | 2.23E-05 | 2.06E-04 | 9.12E-06 | 1.41E-04 | 4.24E-05 | 27 | 1.47E-04 | 4.50E-05 | 1.63E-04 | 3.10E-05 | 1.49E-04 | 2.29E-06 |
| 30 | 1.74E-04 | 1.39E-05 | 1.53E-04 | 8.68E-05 | 2.95E-04 | 1.75E-05 | 30 | 6.33E-05 | 2.61E-06 | 1.88E-04 | 1.02E-05 | 1.81E-04 | 9.15E-05 | 30 | 1.17E-04 | 7.43E-06 | 1.45E-04 | 8.46E-05 | 1.43E-04 | 1.02E-05 |
| 33 | 1.37E-04 | 3.29E-05 | 1.43E-04 | 6.06E-05 | 3.08E-04 | 1.70E-05 | 33 | 1.41E-04 | 5.86E-05 | 2.41E-04 | 3.04E-05 | 1.50E-04 | 6.65E-05 | 33 | 1.52E-04 | 2.40E-05 | 1.69E-04 | 8.65E-05 | 1.43E-04 | 1.57E-05 |
| 36 | 1.58E-04 | 6.03E-05 | 1.02E-04 | 4.24E-05 | 2.70E-04 | 5.19E-09 | 36 | 1.01E-04 | 1.36E-05 | 1.99E-04 | 1.24E-05 | 1.47E-04 | 8.51E-06 | 36 | 2.11E-04 | 2.30E-05 | 1.35E-04 | 4.70E-05 | 1.36E-04 | 2.23E-05 |
| 39 | 1.05E-04 | 8.19E-06 | 1.36E-04 | 4.05E-05 | 2.96E-04 | 2.81E-05 | 39 | 6.07E-05 | 1.18E-05 | 2.11E-04 | 1.97E-05 | 1.50E-04 | 5.87E-05 | 39 | 1.44E-04 | 6.32E-07 | 1.38E-04 | 8.89E-05 | 1.53E-04 | 1.07E-05 |
| 42 | 1.04E-04 | 4.16E-06 | 2.30E-04 | 1.95E-05 | 2.82E-04 | 6.77E-06 | 42 | 9.33E-05 | 1.57E-05 | 2.25E-04 | 2.03E-05 | 1.60E-04 | 5.65E-05 | 42 | 1.39E-04 | 6.20E-06 | 1.70E-04 | 9.24E-05 | 1.73E-04 | 9.23E-06 |
| 45 | 1.04E-04 | 2.43E-06 | 2.70E-04 | 7.22E-06 | 3.16E-04 | 2.98E-07 | 45 | 1.36E-04 | 3.25E-05 | 3.17E-04 | 6.39E-05 | 2.24E-04 | 8.52E-05 | 45 | 1.28E-04 | 2.84E-05 | 1.71E-04 | 6.67E-05 | 1.54E-04 | 8.53E-06 |
| 48 | 8.48E-05 | 1.29E-05 | 1.95E-04 | 4.20E-05 | 3.04E-04 | 7.97E-07 | 48 | 6.19E-05 | 1.55E-05 | 2.28E-04 | 8.40E-06 | 1.20E-04 | 2.23E-05 | 48 | 1.34E-04 | 1.59E-05 | 1.38E-04 | 9.19E-05 | 1.44E-04 | 6.11E-06 |
| 51 | 7.90E-05 | 2.15E-06 | 2.84E-04 | 1.12E-05 | 2.86E-04 | 8.96E-06 | 51 | 1.01E-04 | 2.11E-05 | 2.36E-04 | 4.35E-05 | 1.63E-04 | 4.77E-06 | 51 | 1.63E-04 | 1.76E-05 | 1.36E-04 | 5.64E-05 | 1.50E-04 | 2.08E-05 |
| 54 | 9.34E-05 | 2.30E-06 | 2.44E-04 | 4.49E-05 | 3.12E-04 | 2.39E-05 | 54 | 5.88E-05 | 1.39E-05 | 2.47E-04 | 1.96E-05 | 1.82E-04 | 7.30E-05 | 54 | 1.39E-04 | 9.91E-06 | 1.36E-04 | 9.16E-05 | 1.56E-04 | 5.19E-06 |
| 57 | 8.52E-05 | 7.18E-06 | 1.73E-04 | 1.26E-05 | 2.74E-04 | 4.83E-06 | 57 | 7.02E-05 | 5.25E-06 | 2.45E-04 | 4.10E-06 | 1.92E-04 | 1.83E-05 | 57 | 1.30E-04 | 2.05E-06 | 1.59E-04 | 9.37E-05 | 1.87E-04 | 4.33E-06 |
| 60 | 9.90E-05 | 8.85E-06 | 7.32E-05 | 1.42E-05 | 2.94E-04 | 2.79E-05 | 60 | 7.37E-05 | 1.07E-05 | 2.77E-04 | 4.07E-06 | 1.97E-04 | 6.32E-05 | 60 | 9.52E-05 | 9.17E-06 | 1.78E-04 | 1.15E-04 | 1.54E-04 | 2.16E-05 |

**The Data of Figure 2**

*D*: Runoff depth (m).

**The Data of Figure 2**

| Rainfall intensity  (mm h-1)-Slope (°) | 45-5 | | 90-5 | | 120-5 | |  | 45-10 | | 90-10 | | 120-10 | |  | 45-15 | | 90-15 | | 120-15 | |
| --- | --- | --- | --- | --- | --- | --- | --- | --- | --- | --- | --- | --- | --- | --- | --- | --- | --- | --- | --- | --- |
| Time (*min*) | τ | STDVE | τ | STDVE | τ | STDVE | Time (t) | τ | STDVE | τ | STDVE | τ | STDVE | Time (t) | τ | STDVE | τ | STDVE | τ | STDVE |
| 3 | 0.08242 | 0.00352 | 0.17002 | 0.06029 | 0.22054 | 0.06184 | 3 | 0.2562 | 0.03041 | 0.26201 | 0.00789 | 0.16478 | 0.04477 | 3 | 0.25497 | 0.03884 | 0.31014 | 0.03298 | 0.32594 | 0.02792 |
| 6 | 0.11614 | 0.01098 | 0.1395 | 0.0134 | 0.21368 | 0.00198 | 6 | 0.20006 | 0.04918 | 0.35955 | 0.07613 | 0.13823 | 0.00434 | 6 | 0.42531 | 0.00905 | 0.30089 | 0.07162 | 0.31155 | 0.06375 |
| 9 | 0.17329 | 0.02607 | 0.19737 | 0.04182 | 0.19537 | 0.02494 | 9 | 0.23477 | 0.01291 | 0.29418 | 0.00498 | 0.27171 | 0.16918 | 9 | 0.42227 | 0.01254 | 0.3809 | 0.00452 | 0.37195 | 0.01613 |
| 12 | 0.11177 | 0.01416 | 0.10685 | 0.00454 | 0.20816 | 0.01299 | 12 | 0.14698 | 0.01311 | 0.35826 | 0.04341 | 0.15434 | 0.01339 | 12 | 0.37569 | 0.07031 | 0.30425 | 0.10083 | 0.36738 | 0.05428 |
| 15 | 0.1504 | 0.00293 | 0.15254 | 0.02264 | 0.23335 | 0.00931 | 15 | 0.20907 | 0.0735 | 0.29125 | 0.01994 | 0.16876 | 0.03347 | 15 | 0.24191 | 0.03707 | 0.37293 | 0.16692 | 0.37696 | 0.05257 |
| 18 | 0.17079 | 0.01126 | 0.10482 | 0.00172 | 0.25019 | 0.01342 | 18 | 0.23108 | 0.01448 | 0.43125 | 0.04156 | 0.29228 | 0.11574 | 18 | 0.25357 | 0.08561 | 0.44553 | 0.11637 | 0.41342 | 0.00663 |
| 21 | 0.14922 | 0.0019 | 0.11758 | 0.00759 | 0.21716 | 0.00711 | 21 | 0.13555 | 0.00892 | 0.36171 | 0.08831 | 0.2611 | 0.08198 | 21 | 0.37335 | 0.06414 | 0.32281 | 0.13038 | 0.32081 | 0.04359 |
| 24 | 0.13724 | 0.00445 | 0.09098 | 0.00494 | 0.26542 | 1.90E-04 | 24 | 0.21227 | 0.10534 | 0.34026 | 0.00625 | 0.29229 | 0.15636 | 24 | 0.36707 | 0.09817 | 0.41251 | 0.25001 | 0.3391 | 0.0315 |
| 27 | 0.13974 | 0.0098 | 0.10297 | 0.02214 | 0.23759 | 0.02537 | 27 | 0.26708 | 0.0386 | 0.35568 | 0.01576 | 0.24312 | 0.07331 | 27 | 0.38562 | 0.11805 | 0.42873 | 0.08152 | 0.39179 | 0.00603 |
| 30 | 0.14921 | 0.01191 | 0.13146 | 0.07442 | 0.25272 | 0.01499 | 30 | 0.10934 | 0.0045 | 0.32566 | 0.01759 | 0.31196 | 0.1582 | 30 | 0.30681 | 0.01952 | 0.38142 | 0.22211 | 0.37428 | 0.02687 |
| 33 | 0.1172 | 0.02823 | 0.12303 | 0.05193 | 0.26406 | 0.01459 | 33 | 0.24356 | 0.10127 | 0.4168 | 0.05249 | 0.25857 | 0.11486 | 33 | 0.39915 | 0.06309 | 0.44333 | 0.2271 | 0.37652 | 0.04112 |
| 36 | 0.13509 | 0.05168 | 0.08725 | 0.03635 | 0.23113 | 4.45E-06 | 36 | 0.17482 | 0.02343 | 0.34386 | 0.02147 | 0.25372 | 0.01471 | 36 | 0.55345 | 0.06041 | 0.35355 | 0.12349 | 0.35585 | 0.05845 |
| 39 | 0.08974 | 0.00702 | 0.11626 | 0.03476 | 0.25349 | 0.02413 | 39 | 0.10488 | 0.02046 | 0.36387 | 0.03404 | 0.25901 | 0.10145 | 39 | 0.37887 | 0.00166 | 0.36167 | 0.2334 | 0.40077 | 0.02812 |
| 42 | 0.08884 | 0.00357 | 0.19686 | 0.01669 | 0.24142 | 0.0058 | 42 | 0.16115 | 0.02705 | 0.38834 | 0.03512 | 0.27633 | 0.09767 | 42 | 0.36562 | 0.01627 | 0.44641 | 0.24263 | 0.45333 | 0.02424 |
| 45 | 0.08895 | 0.00208 | 0.23171 | 0.00619 | 0.27104 | 2.55E-04 | 45 | 0.2358 | 0.05612 | 0.5477 | 0.11048 | 0.38632 | 0.14721 | 45 | 0.33639 | 0.0747 | 0.44817 | 0.17513 | 0.40536 | 0.02241 |
| 48 | 0.07269 | 0.01104 | 0.16707 | 0.03603 | 0.26094 | 6.83E-04 | 48 | 0.10701 | 0.02676 | 0.3936 | 0.01451 | 0.20753 | 0.03859 | 48 | 0.35151 | 0.0418 | 0.36325 | 0.2413 | 0.37898 | 0.01604 |
| 51 | 0.06775 | 0.00184 | 0.24327 | 0.00956 | 0.24483 | 0.00768 | 51 | 0.17473 | 0.03641 | 0.40806 | 0.07522 | 0.28208 | 0.00824 | 51 | 0.42708 | 0.04634 | 0.35835 | 0.14816 | 0.39415 | 0.05461 |
| 54 | 0.08004 | 0.00197 | 0.20911 | 0.03854 | 0.26755 | 0.02046 | 54 | 0.10157 | 0.02399 | 0.42656 | 0.03382 | 0.31433 | 0.12609 | 54 | 0.36499 | 0.02602 | 0.35833 | 0.24044 | 0.41071 | 0.01364 |
| 57 | 0.07303 | 0.00615 | 0.14833 | 0.0108 | 0.23507 | 0.00414 | 57 | 0.12128 | 0.00907 | 0.42296 | 0.00708 | 0.33173 | 0.0317 | 57 | 0.34057 | 0.00538 | 0.41656 | 0.24604 | 0.49216 | 0.01137 |
| 60 | 0.08489 | 0.00759 | 0.06278 | 0.01217 | 0.25214 | 0.02394 | 60 | 0.12728 | 0.01845 | 0.4791 | 0.00703 | 0.34038 | 0.10922 | 60 | 0.24991 | 0.02409 | 0.4679 | 0.30252 | 0.40472 | 0.05664 |

τ: Shear stress (Pa).

**The Data of Figure 2**

| Rainfall intensity  (mm h-1)-Slope (°) | 45-5 | | 90-5 | | 120-5 | |  | 45-10 | | 90-10 | | 120-10 | |  | 45-15 | | 90-15 | | 120-15 | |
| --- | --- | --- | --- | --- | --- | --- | --- | --- | --- | --- | --- | --- | --- | --- | --- | --- | --- | --- | --- | --- |
| Time (*min*) | ω | STDVE | ω | STDVE | ω | STDVE | Time (t) | ω | STDVE | ω | STDVE | ω | STDVE | Time (t) | ω | STDVE | ω | STDVE | ω | STDVE |
| 3 | 0.00532 | 9.07E-04 | 0.01097 | 0.00187 | 0.01418 | 0.00325 | 3 | 0.00896 | 8.93E-05 | 0.03054 | 9.20E-04 | 0.01627 | 0.00151 | 3 | 0.01554 | 1.23E-04 | 0.045 | 0.00446 | 0.06208 | 0.00255 |
| 6 | 0.00407 | 3.66E-04 | 0.01242 | 0.0014 | 0.0154 | 6.34E-04 | 6 | 0.00922 | 5.73E-04 | 0.02983 | 0.00143 | 0.01545 | 0.00112 | 6 | 0.01665 | 6.99E-04 | 0.03538 | 0.00329 | 0.06397 | 0.00497 |
| 9 | 0.00467 | 2.31E-04 | 0.0123 | 0.00172 | 0.01585 | 9.88E-04 | 9 | 0.00977 | 6.14E-04 | 0.02923 | 0.00122 | 0.0261 | 0.00658 | 9 | 0.01762 | 5.01E-04 | 0.04204 | 1.20E-04 | 0.06541 | 0.00498 |
| 12 | 0.00427 | 7.29E-05 | 0.01218 | 0.00211 | 0.01617 | 0.00108 | 12 | 0.0096 | 6.38E-04 | 0.03027 | 6.26E-04 | 0.01627 | 6.06E-04 | 12 | 0.01789 | 3.56E-04 | 0.03407 | 0.00457 | 0.06306 | 0.00496 |
| 15 | 0.00448 | 2.01E-04 | 0.01143 | 0.00259 | 0.01609 | 9.83E-04 | 15 | 0.01001 | 6.57E-04 | 0.03033 | 9.72E-04 | 0.01544 | 8.92E-04 | 15 | 0.01832 | 3.65E-05 | 0.03038 | 0.0068 | 0.06443 | 0.00497 |
| 18 | 0.00475 | 1.11E-04 | 0.0109 | 0.00285 | 0.01675 | 7.53E-04 | 18 | 0.01029 | 4.16E-04 | 0.03102 | 6.25E-04 | 0.02687 | 0.00591 | 18 | 0.0185 | 3.88E-05 | 0.04077 | 0.00132 | 0.06502 | 0.00511 |
| 21 | 0.00457 | 7.52E-05 | 0.01064 | 0.00342 | 0.01678 | 8.73E-04 | 21 | 0.01006 | 6.39E-04 | 0.03107 | 5.77E-04 | 0.02733 | 0.00505 | 21 | 0.01841 | 3.05E-04 | 0.02858 | 0.00678 | 0.06214 | 0.00439 |
| 24 | 0.00462 | 5.85E-06 | 0.0103 | 0.00381 | 0.01671 | 0.00109 | 24 | 0.0101 | 8.04E-04 | 0.03079 | 6.60E-04 | 0.02706 | 0.00532 | 24 | 0.01873 | 1.52E-04 | 0.02889 | 0.00728 | 0.06388 | 0.00447 |
| 27 | 0.00469 | 1.20E-04 | 0.00999 | 0.00392 | 0.01704 | 0.0011 | 27 | 0.00999 | 6.62E-04 | 0.03052 | 4.89E-04 | 0.02796 | 0.00571 | 27 | 0.0184 | 4.90E-05 | 0.03591 | 0.00397 | 0.06394 | 0.00387 |
| 30 | 0.00473 | 7.62E-05 | 0.00942 | 0.00443 | 0.01684 | 0.00111 | 30 | 0.00981 | 5.44E-04 | 0.02904 | 2.96E-04 | 0.02792 | 0.00539 | 30 | 0.01897 | 2.59E-04 | 0.02911 | 0.00664 | 0.06488 | 0.00429 |
| 33 | 0.00492 | 5.07E-05 | 0.01085 | 0.00614 | 0.01676 | 8.35E-04 | 33 | 0.01022 | 3.68E-04 | 0.0324 | 5.55E-04 | 0.02833 | 0.00464 | 33 | 0.01887 | 4.86E-05 | 0.02724 | 0.00714 | 0.06565 | 0.00527 |
| 36 | 0.0062 | 1.67E-04 | 0.00699 | 0.004 | 0.01675 | 0.00103 | 36 | 0.01036 | 7.45E-05 | 0.03012 | 4.14E-04 | 0.03595 | 4.18E-04 | 36 | 0.01882 | 3.84E-04 | 0.02927 | 0.00557 | 0.06499 | 0.00471 |
| 39 | 0.00609 | 4.27E-04 | 0.01019 | 0.00372 | 0.01717 | 0.00109 | 39 | 0.0106 | 3.56E-04 | 0.03037 | 8.15E-04 | 0.02774 | 0.0036 | 39 | 0.0189 | 1.49E-04 | 0.02391 | 0.0076 | 0.06443 | 0.00389 |
| 42 | 0.00597 | 3.75E-04 | 0.01469 | 4.52E-04 | 0.01674 | 0.00107 | 42 | 0.01093 | 5.55E-04 | 0.03051 | 0.00114 | 0.02802 | 0.0043 | 42 | 0.01848 | 7.52E-05 | 0.02911 | 0.00566 | 0.06511 | 0.00477 |
| 45 | 0.006 | 3.60E-04 | 0.01431 | 4.70E-04 | 0.01669 | 0.00101 | 45 | 0.01101 | 5.02E-04 | 0.03063 | 0.00167 | 0.03434 | 0.0012 | 45 | 0.01882 | 4.25E-05 | 0.02797 | 0.00673 | 0.06351 | 0.00474 |
| 48 | 0.00582 | 1.62E-04 | 0.0155 | 1.94E-04 | 0.01679 | 0.00101 | 48 | 0.01064 | 4.54E-04 | 0.02985 | 0.00139 | 0.02726 | 0.00447 | 48 | 0.01887 | 2.09E-04 | 0.02416 | 0.008 | 0.06361 | 0.00476 |
| 51 | 0.00592 | 1.49E-04 | 0.01619 | 3.35E-04 | 0.01699 | 0.00101 | 51 | 0.01096 | 6.88E-04 | 0.02988 | 7.67E-04 | 0.02915 | 0.00388 | 51 | 0.01901 | 1.97E-04 | 0.0271 | 0.00678 | 0.06423 | 0.00413 |
| 54 | 0.00594 | 2.06E-04 | 0.01616 | 2.19E-04 | 0.01686 | 0.00102 | 54 | 0.01107 | 4.71E-04 | 0.02965 | 5.58E-04 | 0.02836 | 0.00387 | 54 | 0.01898 | 6.77E-05 | 0.0253 | 0.00746 | 0.0634 | 0.00443 |
| 57 | 0.00616 | 9.36E-05 | 0.01069 | 1.93E-04 | 0.01677 | 9.92E-04 | 57 | 0.0112 | 5.38E-04 | 0.03061 | 0.00153 | 0.03637 | 6.25E-04 | 57 | 0.01879 | 2.76E-04 | 0.02552 | 0.00764 | 0.06485 | 0.00506 |
| 60 | 0.00628 | 1.02E-04 | 0.00543 | 1.31E-04 | 0.0164 | 0.00186 | 60 | 0.01108 | 5.18E-04 | 0.02939 | 8.81E-04 | 0.02898 | 0.00314 | 60 | 0.01185 | 3.79E-04 | 0.02729 | 0.00613 | 0.06156 | 0.00651 |

ω: Stream power (W m^−2^).

**The Data of Figure 3**

| Rainfall intensity (mm h^-1^)-Slope (°) | <0.002 | | 0.002-0.02 | | 0.02-0.05 | | 0.05-0.25 | | >0.25 |
| --- | --- | --- | --- | --- | --- | --- | --- | --- | --- |
|  | P | E/D-average | P | E/D-average | P | E/D-average | P | E/D-average | P |
| 45-5 | 49.65737 | 1.019803 | 40.1205 | 1.064146 | 8.084121 | 0.865355 | 2.138006 | 0.551525 | 0 |
| 45-10 | 38.09404 | 0.874617 | 34.26676 | 1.004427 | 18.41187 | 1.05113 | 9.060662 | 1.762121 | 0.166667 |
| 45-15 | 31.17225 | 0.82173 | 35.32821 | 0.960512 | 21.59028 | 1.09396 | 11.71164 | 2.344658 | 0.197624 |
| 90-5 | 32.96689 | 0.844018 | 37.34024 | 0.944327 | 18.84243 | 1.164531 | 8.198253 | 1.924737 | 2.652195 |
| 90-10 | 25.30186 | 0.769666 | 33.88079 | 0.936725 | 25.85161 | 1.110558 | 13.01157 | 1.915652 | 1.954177 |
| 90-15 | 21.08753 | 0.654743 | 28.95706 | 0.843421 | 28.51376 | 1.124579 | 18.17624 | 2.591281 | 3.265414 |
| 120-5 | 30.7899 | 0.861229 | 34.59885 | 0.931477 | 21.54874 | 1.059113 | 9.790097 | 1.62396 | 3.272411 |
| 120-10 | 22.55962 | 0.737024 | 28.40921 | 0.809058 | 26.7191 | 0.976385 | 17.17436 | 2.801811 | 5.137712 |
| 120-15 | 21.05383 | 0.65865 | 28.27442 | 0.794388 | 26.83305 | 1.044829 | 16.44418 | 2.792894 | 7.39451 |

*P*: Percentage of corresponding size class sediment particles;

*E/D*: The ratios of effective/ultimate size class sediment particles.

**The Data of Figure 4**

| Rainfall intensity (mm h^-1^)-Slope (°) | *V* | *D* | Der (0.05-0.25 mm) | Der (>0.25mm) |
| --- | --- | --- | --- | --- |
| 45-5 | 0.05287 | 0.01329 | 1.78066 | 0 |
| 45-10 | 0.06745 | 0.01028 | 3.84186 | 0.16667 |
| 45-15 | 0.05351 | 0.01366 | 6.74568 | 0.17763 |
| 90-5 | 0.08351 | 0.01691 | 3.78861 | 2.483 |
| 90-10 | 0.08328 | 0.02191 | 6.32035 | 1.42979 |
| 90-15 | 0.08463 | 0.02096 | 10.94107 | 2.57555 |
| 120-5 | 0.06918 | 0.02808 | 3.74395 | 2.73622 |
| 120-10 | 0.10707 | 0.01507 | 10.96753 | 4.71457 |
| 120-15 | 0.17006 | 0.0146 | 10.34146 | 6.90113 |

*V*: Flow velocity (m s^−1^);

*D*: Runoff depth (m).

**The Data of Figure 5**

| Rainfall intensity (mm h^−1^)-Slope (°) | *V* | *D* | *ERoc* of > 2 mm | *ERoc* of 0.25-2 mm | *ERoc* of 0.05-0.25 mm | *ERoc* of <0.05 mm | Total *ERoc* |
| --- | --- | --- | --- | --- | --- | --- | --- |
| 45-5 | 0.05287 | 0.01329 | 1 | 1 | 3.5 | 2.16822 | 2.36396 |
| 45-10 | 0.06745 | 0.01028 | 1.37029 | 2.54631 | 1.43546 | 1.42337 | 1.95844 |
| 45-15 | 0.05351 | 0.01366 | 0.99511 | 1.59639 | 1.44416 | 1.53654 | 1.81276 |
| 90-5 | 0.08351 | 0.01691 | 1 | 2.58129 | 1.46295 | 1.413 | 1.78547 |
| 90-10 | 0.08328 | 0.02191 | 1.15686 | 1.52191 | 1.32322 | 1.25568 | 1.56319 |
| 90-15 | 0.08463 | 0.02096 | 0.9009 | 1.40906 | 1.25299 | 1.21026 | 1.59685 |
| 120-5 | 0.06918 | 0.02808 | 0.90903 | 2.46047 | 2.2629 | 1.47611 | 1.92446 |
| 120-10 | 0.10707 | 0.01507 | 0.68251 | 1.12756 | 1.11188 | 1.16028 | 1.15901 |
| 120-15 | 0.17006 | 0.0146 | 0.96587 | 1.39701 | 1.23055 | 1.23652 | 1.51963 |

**The Data of Figure 6**

| **45 mm/h-5°** | SOC concentration (g kg^−1^) | | | | **45 mm/h-10°** | SOC concentration (g kg^−1^) | | | | **45 mm/h-15°** | SOC concentration (g kg^−1^) | | | |
| --- | --- | --- | --- | --- | --- | --- | --- | --- | --- | --- | --- | --- | --- | --- |
| Time (min) | > 2mm | 2-0.25 mm | 0.25-0.05 mm | <0.05 mm | Time (min) | > 2mm | 2-0.25 mm | 0.25-0.05 mm | <0.05 mm | Time (min) | > 2mm | 2-0.25 mm | 0.25-0.05 mm | <0.05 mm |
| 12 | -- | -- | -- | 13.18748 | 12 | -- | 17.5877 | 12.84743 | 8.89783 | 12 | 14.97086 | 18.2277 | 18.20898 | 8.55878 |
| 24 | -- | -- | -- | 12.3529 | 24 | 17.21123 | 17.26597 | 13.42922 | 7.38399 | 24 | 11.97368 | 15.46193 | 14.00504 | 7.08042 |
| 36 | -- | -- | -- | 12.40798 | 36 | -- | 21.33265 | 15.76076 | 7.37671 | 36 | -- | -- | 10.99407 | 9.39662 |
| 48 | -- | -- | 39.54633 | 10.24056 | 48 | 18.68253 | 19.77377 | 15.55826 | 7.13221 | 48 | -- | 15.88992 | 16.48641 | 7.81283 |
| 60 | -- | -- | 32.02531 | 9.63561 | 60 | 19.76099 | 22.89176 | 15.63815 | 7.16916 | 60 | -- |  | 13.98267 | 8.12949 |
| Average |  |  | 35.78582 | 11.56491 | Average | 18.55158 | 19.77037 | 14.64676 | 7.59198 | Average | 13.47227 | 16.52652 | 14.73543 | 8.195628 |
| **90 mm/h-5°** | SOC concentration (g kg^−1^) | | | | **90 mm/h-10°** | SOC concentration (g kg^−1^) | | | | **90 mm/h-15°** | SOC concentration (g kg^−1^) | | | |
| Time (min) | > 2mm | 2-0.25 mm | 0.25-0.05 mm | <0.05 mm | Time (min) | > 2mm | 2-0.25 mm | 0.25-0.05 mm | <0.05 mm | Time (min) | > 2mm | 2-0.25 mm | 0.25-0.05 mm | <0.05 mm |
| 12 | -- | 27.5606 | 14.01023 | 9.26568 | 12 | 14.32604 | 14.96441 | 14.01823 | 6.52726 | 12 | 9.13472 | 12.48198 | 11.79141 | 6.29695 |
| 24 | -- | 20.666 | 15.20981 | 7.33899 | 24 | 18.30208 | 12.0001 | 12.59445 | 6.6554 | 24 | 10.51025 | 11.0052 | 13.40214 | 6.65084 |
| 36 | -- | 17.70324 | 14.93405 | 6.97468 | 36 | 10.07549 | 11.37709 | 14.17697 | 6.83879 | 36 | 14.24976 | 10.29785 | 12.99204 | 6.50572 |
| 48 | -- | 17.90105 | 15.79607 | 6.92185 | 48 | 19.13629 | 10.58695 | 12.33367 | 6.28843 | 48 | 12.17273 | 10.69396 | 12.55877 | 6.57559 |
| 60 | -- | 16.37896 | 14.68583 | 7.18216 | 60 | 16.47015 | 10.15457 | 14.38423 | 7.1779 | 60 | 14.9162 | 10.22313 | 13.17984 | 6.24753 |
| Average |  | 20.04197 | 14.9272 | 7.536672 | Average | 15.66201 | 11.81662 | 13.50151 | 6.697556 | Average | 12.19673 | 10.94042 | 12.78484 | 6.455326 |
| **120 mm/h-5°** | SOC concentration (g kg^−1^) | | | | **120 mm/h-10°** | SOC concentration (g kg^−1^) | | | | **120 mm/h-15°** | SOC concentration (g kg^−1^) | | | |
| Time (min) | > 2mm | 2-0.25 mm | 0.25-0.05 mm | <0.05 mm | Time (min) | > 2mm | 2-0.25 mm | 0.25-0.05 mm | <0.05 mm | Time (min) | > 2mm | 2-0.25 mm | 0.25-0.05 mm | <0.05 mm |
| 12 | -- | 25.37706 | 30.52058 | 8.74234 | 12 | 9.44369 | 9.06139 | 8.16132 | 6.34237 | 12 | 19.54416 | 11.66947 | 12.54184 | 5.95602 |
| 24 | 12.32475 | 20.23117 | 22.14541 | 8.23251 | 24 | 10.93128 | 9.01759 | 11.75477 | 6.0594 | 24 | 11.96951 | 10.65073 | 13.38548 | 6.07612 |
| 36 | 13.72925 | 21.2979 | 24.11915 | 7.5959 | 36 | 9.08342 | 8.75611 | 12.84978 | 6.04316 | 36 | 11.62168 | 10.34154 | 12.69754 | 6.1494 |
| 48 | 8.74645 | 15.53862 | 20.11796 | 7.434 | 48 | 8.90008 | 8.94828 | 12.43914 | 6.25544 | 48 | 12.17641 | 9.80447 | 13.48011 | 9.10669 |
| 60 | 14.42673 | 13.07474 | 18.54424 | 7.36162 | 60 | 7.84183 | 7.99012 | 11.52047 | 6.2431 | 60 | 10.07007 | 11.76783 | 10.67439 | 5.68865 |
| Average | 12.3068 | 19.1039 | 23.08947 | 7.873274 | Average | 9.24006 | 8.754698 | 11.3451 | 6.188694 | Average | 13.07637 | 10.84681 | 12.55587 | 6.595376 |

**The Data of Figure 6**

| Time (min) | Stream power of different treatments (Rainfall intensity (mm h^−1^)-Slope (°)) | | | | | | | | |
| --- | --- | --- | --- | --- | --- | --- | --- | --- | --- |
|  | 45-5 | 90-5 | 120-5 | 45-10 | 90-10 | 120-10 | 45-15 | 90-15 | 120-15 |
| 12 | 0.00427 | 0.0096 | 0.01789 | 0.01218 | 0.03027 | 0.04554 | 0.01617 | 0.01627 | 0.06306 |
| 24 | 0.00462 | 0.0101 | 0.01873 | 0.0103 | 0.03079 | 0.0474 | 0.01671 | 0.02706 | 0.06388 |
| 36 | 0.0062 | 0.01036 | 0.01882 | 0.00699 | 0.03012 | 0.04292 | 0.01675 | 0.03595 | 0.06499 |
| 48 | 0.00582 | 0.01064 | 0.01887 | 0.0155 | 0.02985 | 0.04196 | 0.01679 | 0.02726 | 0.06361 |
| 60 | 0.00628 | 0.01108 | 0.01185 | 0.00543 | 0.02939 | 0.04253 | 0.0164 | 0.02898 | 0.06156 |

**The Data of Figure 7**

| **45 mm/h-5°** | | | | | | **45 mm/h-10°** | | | | | | **45 mm/h-15°** | | | | | |
| --- | --- | --- | --- | --- | --- | --- | --- | --- | --- | --- | --- | --- | --- | --- | --- | --- | --- |
| Time (min) | Ca (%)-A | Ca (%)-B | Ca-Average | STDVE | Sa (%)=100-Ca | Time (min) | Ca (%)-A | Ca (%)-B | Ca-Average | STDVE | Sa (%)=100-Ca | Time (min) | Ca (%)-A | Ca (%)-B | Ca-Average | STDVE | Sa (%)=100-Ca |
| 12-average | 100.00 | 99.70 | 99.85 | 0.14 | 0.15 | 12-average | 72.12 | 90.36 | 81.52 | 9.12 | 18.48 | 12-average | 86.08 | 84.25 | 85.14 | 0.91 | 14.86 |
| 24-average | 93.80 | 98.00 | 95.90 | 1.63 | 4.10 | 24-average | 68.63 | 73.65 | 70.94 | 2.52 | 29.06 | 24-average | 71.42 | 71.65 | 71.54 | 0.11 | 28.46 |
| 36-average | 94.89 | 95.00 | 94.95 | 0.00 | 5.05 | 36-average | 64.04 | 68.29 | 66.46 | 2.13 | 33.54 | 36-average | 79.54 | 82.97 | 81.22 | 1.72 | 18.78 |
| 48-average | 89.24 | 90.93 | 90.08 | 0.76 | 9.92 | 48-average | 75.93 | 72.94 | 74.39 | 1.50 | 25.61 | 48-average | 78.54 | 80.09 | 79.28 | 0.78 | 20.72 |
| 60-average | 80.05 | 100.00 | 90.03 | 8.14 | 9.97 | 60-average | 74.98 | 74.17 | 74.56 | 0.40 | 25.44 | 60-average | 72.98 | 74.00 | 73.48 | 0.51 | 26.52 |
| total-average | 91.60 | 96.73 | 94.16 | 2.14 | 5.84 | total-average | 71.14 | 75.88 | 73.57 | 3.13 | 26.43 | total-average | 77.71 | 78.59 | 78.13 | 0.80 | 21.87 |
| **90 mm/h-5°** | | | | | | **90 mm/h-10°** | | | | | | **90 mm/h-15°** | | | | | |
| Time (min) | Ca (%)-A | Ca (%)-B | Ca-Average | STDVE | Sa (%)=100-Ca | Time (min) | Ca (%)-A | Ca (%)-B | Ca-Average | STDVE | Sa (%)=100-Ca | Time (min) | Ca (%)-A | Ca (%)-B | Ca-Average | STDVE | Sa (%)=100-Ca |
| 12-average | 100.00 | 96.87 | 98.43 | 3.60 | 1.57 | 12-average | 81.11 | 68.04 | 72.62 | 6.63 | 27.38 | 12-average | 46.46 | 49.76 | 48.14 | 1.65 | 51.86 |
| 24-average | 82.28 | 77.94 | 80.15 | 2.17 | 19.85 | 24-average | 50.15 | 59.97 | 54.48 | 4.92 | 45.52 | 24-average | 59.52 | 57.91 | 58.68 | 0.80 | 41.32 |
| 36-average | 83.14 | 77.62 | 80.23 | 2.76 | 19.77 | 36-average | 66.54 | 63.58 | 64.96 | 1.48 | 35.04 | 36-average | 53.45 | 53.14 | 53.30 | 0.16 | 46.70 |
| 48-average | 83.31 | 78.45 | 80.78 | 2.43 | 19.22 | 48-average | 58.16 | 52.22 | 54.89 | 2.97 | 45.11 | 48-average | 57.12 | 52.74 | 54.81 | 2.19 | 45.19 |
| 60-average | 78.19 | 80.61 | 79.27 | 1.21 | 20.73 | 60-average | 76.51 | 57.86 | 64.97 | 9.41 | 35.03 | 60-average | 53.91 | 49.45 | 51.44 | 2.23 | 48.56 |
| total-average | 85.38 | 82.30 | 84.18 | 2.43 | 16.23 | total-average | 66.49 | 60.33 | 62.38 | 5.08 | 37.62 | total-average | 54.09 | 52.60 | 53.27 | 1.41 | 46.73 |
| **120 mm/h-5°** | | | | | | **120 mm/h-10°** | | | | | | **120 mm/h-15°** | | | | | |
| Time (min) | Ca (%)-A | Ca (%)-B | Ca-Average | STDVE | Sa (%)=100-Ca | Time (min) | Ca (%)-A | Ca (%)-B | Ca-Average | STDVE | Sa (%)=100-Ca | Time (min) | Ca (%)-A | Ca (%)-B | Ca-Average | STDVE | Sa (%)=100-Ca |
| 12-average | 101.63 | 97.33 | 99.25 | 2.15 | 0.75 | 12-average | 116.54 | 93.06 | 104.02 | 11.75 | -4.02 | 12-average | 59.57 | 57.43 | 58.37 | 1.07 | 41.63 |
| 24-average | 85.37 | 87.94 | 86.64 | 1.28 | 13.36 | 24-average | 124.03 | 66.97 | 85.75 | 29.08 | 14.25 | 24-average | 57.66 | 56.06 | 56.83 | 0.80 | 43.17 |
| 36-average | 86.53 | 86.33 | 86.42 | 0.10 | 13.58 | 36-average | 106.59 | 44.22 | 59.45 | 32.52 | 40.55 | 36-average | 53.47 | 51.18 | 52.24 | 1.15 | 47.76 |
| 48-average | 87.05 | 82.07 | 84.33 | 2.49 | 15.67 | 48-average | 108.49 | 46.02 | 63.72 | 32.20 | 36.28 | 48-average | 74.42 | 68.96 | 71.63 | 2.73 | 28.37 |
| 60-average | 79.76 | 76.55 | 78.07 | 1.60 | 21.93 | 60-average | 31.16 | 107.92 | 53.24 | 39.52 | 46.76 | 60-average | 46.05 | 47.93 | 47.08 | 0.94 | 52.92 |
| total-average | 88.07 | 86.05 | 86.94 | 1.53 | 13.06 | total-average | 97.36 | 71.64 | 73.24 | 29.01 | 26.76 | total-average | 58.23 | 56.31 | 57.23 | 1.34 | 42.77 |
